# World Heart Federation Roadmap on Atrial Fibrillation – A 2020 Update

**DOI:** 10.5334/gh.1023

**Published:** 2021-05-27

**Authors:** Ben Freedman, Gerhard Hindricks, Amitava Banerjee, Adrian Baranchuk, Chi Keong Ching, Xin Du, Donna Fitzsimons, Jeff S. Healey, Takanori Ikeda, Trudie C. A. Lobban, Amam Mbakwem, Calambur Narasimhan, Lis Neubeck, Peter Noseworthy, Daniel M. Philbin, Fausto J. Pinto, Joselyn Rwebembera, Renate B. Schnabel, Jesper Hastrup Svendsen, Luis Aguinaga, Elena Arbelo, Michael Böhm, Hasan Ali Farhan, F. D. Richard Hobbs, Antoni Martínez-Rubio, Claudio Militello, Nitish Naik, Jean Jacques Noubiap, Pablo Perel, Daniel José Piñeiro, Antonio Luiz Ribeiro, Janina Stepinska

**Affiliations:** 1 Heart Research Institute, University of Sydney, Sydney, AU; 2 Leipzig Heart Institute, Leipzig, DE; 3 University College London, London, UK; 4 Queen’s University, Kingston, CA; 5 National Heart Centre Singapore, SG; 6 Beijing Anzhen Hospital, Capital Medical University, CN; 7 Queen’s University Belfast, UK; 8 McMaster University, Hamilton, Ontario, CA; 9 Toho University Faculty of Medicine, Tokyo, JP; 10 Arrhythmia Alliance & Atrial Fibrillation Association, Stratford Upon Avon, UK; 11 Lagos University Teaching Hospital, Idi Araba, Lagos, NG; 12 AIG Hospitals, Gachibowli, Telangana, IN; 13 Edinburgh Napier University, Edinburgh, UK; 14 Mayo Clinic, Rochester, US; 15 Brown University, Providence, Rhode Island, US; 16 Santa Maria University Hospital (CHULN), CAML, CCUL, Lisboa, PT; 17 Uganda Heart Institute, Kampala, UG; 18 University Heart and Vascular Centre Hamburg, DE; 19 Copenhagen University Hsopital – Rigshospitalet and University of Copenhagen, DK; 20 Centro integral de arritmias Tucuman, Tucuman, AR; 21 Hospital Clinic de Barcelona, Universitat de Barcelona, ES; 22 Universitätsklinikum des Saarlandes, Hornburg/Saar, DE; 23 Baghdad Heart Center, Baghdad, IQ; 24 University of Oxford, Oxford, UK; 25 University Hospital of Sabadell, Universitat Autonoma de Barcelona, ES; 26 Sanatorio Sagrado Corazón. OSECAC. Buenos Aires, AR; 27 All India Institute of Medical Sciences, New Delhi, IN; 28 University of Adelaide, Adelaide, AU; 29 World Heart Federation, Geneva, CH; 30 Universidad de Buenos Aires, AR; 31 Universidade Federal de Minas Gerais, Belo Horizonte, MG, BR; 32 National Institute of Cardiology, Warsaw, PL

**Keywords:** atrial fibrillation, AF, cardiology, WHF, NOACs, digital technology

## Abstract

The World Heart Federation (WHF) commenced a Roadmap initiative in 2015 to reduce the global burden of cardiovascular disease and resultant burgeoning of healthcare costs. Roadmaps provide a blueprint for implementation of priority solutions for the principal cardiovascular diseases leading to death and disability. Atrial fibrillation (AF) is one of these conditions and is an increasing problem due to ageing of the world’s population and an increase in cardiovascular risk factors that predispose to AF. The goal of the AF roadmap was to provide guidance on priority interventions that are feasible in multiple countries, and to identify roadblocks and potential strategies to overcome them.

Since publication of the AF Roadmap in 2017, there have been many technological advances including devices and artificial intelligence for identification and prediction of unknown AF, better methods to achieve rhythm control, and widespread uptake of smartphones and apps that could facilitate new approaches to healthcare delivery and increasing community AF awareness. In addition, the World Health Organisation added the non-vitamin K antagonist oral anticoagulants (NOACs) to the Essential Medicines List, making it possible to increase advocacy for their widespread adoption as therapy to prevent stroke. These advances motivated the WHF to commission a 2020 AF Roadmap update. Three years after the original Roadmap publication, the identified barriers and solutions were judged still relevant, and progress has been slow.

This 2020 Roadmap update reviews the significant changes since 2017 and identifies priority areas for achieving the goals of reducing death and disability related to AF, particularly targeted at low-middle income countries. These include advocacy to increase appreciation of the scope of the problem; plugging gaps in guideline management and prevention through physician education, increasing patient health literacy, and novel ways to increase access to integrated healthcare including mHealth and digital transformations; and greater emphasis on achieving practical solutions to national and regional entrenched barriers. Despite the advances reviewed in this update, the task will not be easy, but the health rewards of implementing solutions that are both innovative and practical will be great.

## Introduction, Context, and Background

About five years ago, the World Heart Federation (WHF) launched a Roadmap initiative to reduce death and disability from non-communicable diseases (NCDs), focusing on the increasing global burden of cardiovascular disease. This rise in cardiovascular disease disproportionately affects people in low- and middle-income countries (LMICs). The aim of this WHF initiative is to reduce death and disability through effective interventions targeting prevention, early detection, and treatment. One of the main problems in achieving the desired outcomes is that the health system resources required to make an impact are most challenging for LMICs, which vary enormously between and within countries in availability of cardiovascular health resources, so it is difficult to generalize. Even in high-income countries (HICs), areas of health inequalities, socioeconomic deprivation, and often poverty are present in many areas and in minorities including first nation peoples (e.g., native Americans and Aboriginal Australians), migrants and different ethnic or racial groups, and may co-exist with greater cardiovascular burdens including atrial fibrillation (AF) and its complications.

The WHF Roadmap for AF was published in December 2017 [1]. The aim of the Roadmap was to ‘provide guidance on priority interventions on a global level that can be adapted to regional contexts’ [1]. The interventions in the Roadmap are intended to be evidence-based, feasible in multiple countries, affordable, and cost-effective. That is a difficult set of conditions to achieve, but one that must be achieved if we are to realise the potentials for prevention of death and suffering related to AF globally. The roadmap identified ideal patient-care pathways with the potential of widespread adoption in multiple national settings. It also identified barriers to implementation of interventions and potential strategies to overcome them.

Much has happened in the three years since publication, including technological advances that will change our ability to detect AF early before it becomes symptomatic, and to better predict those who have silent AF or are likely to soon develop it. There have also been technological advances in reducing AF symptoms through more effective ablation of the arrhythmia, and in prevention of cardio-embolism, but the relevance of these technological advances for LMICs is uncertain. The widespread adoption of mobile technology, particularly smartphones, has also facilitated new approaches to healthcare delivery, which could impact general community awareness of the condition and increase patient knowledge, in turn facilitating adherence to effective lifestyle and pharmacological approaches. Finally, an important outcome of the WHF Emerging Leaders Programme was preparation of a successful submission to the World Health Organization to add the non-vitamin K antagonist (VKA) oral anticoagulants (NOACs) to the Essential Medicines List [2]. These developments prompted the WHF to undertake an update of the AF Roadmap in 2020, focusing on new developments, and an assessment of the status of roadblocks and barriers identified in the original Roadmap.

To accomplish this, an international panel of AF experts was assembled to summarize the areas of the original Roadmap requiring updating – what is new and important for the ideal AF patient-care pathway – and to place this information in perspective. Additionally, an online consultation of national society and foundation representatives was carried out, with respondents from 23 countries. The majority stated that the previously identified roadblocks and the suggested solutions were still relevant in their countries, but it was uncertain how much progress had been made. This update will serve as a vehicle to restate the roadblocks and strategies to overcome them and to examine whether the advances reviewed might provide novel ways to more effectively prevent, detect, treat, and reduce complications associated with AF.

## 1. Global AF Burden and Country Estimates

AF remains the most clinically significant arrhythmia seen in clinical practice [1,3,4,5]. Prevalence is about 1–3% in the general population but rises with age (up to 9%, aged ≥65 years; up to 17%, ≥80 years), presence of comorbidities and male sex, and varies with ethnicity, region, and screening method used [3,4,5,6,7,8,9,10]. Lifetime risk of developing AF also varies with age and ethnicity and is up to 36% for men and 30% for women at age 40–45 and significantly higher in Whites than African Americans [9,11,12]. The burden of AF has been on the increase since 1990 with a doubling of prevalent cases from 1990–2019 [13]. Age-standardized prevalence is similar, suggesting that increases are largely due to ageing of the population and population increase. It is projected that by 2030, 14–17 million people in the EU will be living with AF, representing a doubling of the 2010 global data. For the USA, estimated increase is 12 million [14,15,16]. This increase is attributable to the aging population, increasing AF risk factors and increased screening [13,17,18,19]. Genetic differences affect AF occurrence and may contribute to regional differences in prevalence. European ancestry has been linked to a higher burden of AF [20,21,22], and AF burden is lower in Asians, Hispanics, and Blacks compared to Caucasians [23]. The last global burden of disease surveys showed that AF disability burden was higher in HICs than LMICs [13]. However, this may well be a problem of lower AF ascertainment in many LMICs, as recent studies from Asia using smartphone handheld-electrocardiogram (ECG) technology for community screening have reported increased prevalence compared to earlier studies and some have reported prevalence similar to Europe and USA, Table 1 [19,24,25,26,27,28].

**Table 1:** Temporal trends in country burden of AF detected by AF screening.

Country	Prevalence			

	2001–2010 publications*		2011–2020 publications*	

	Cohort	Burden	Cohort	Burden

Belgium [225]			≥40 years	2.2% (1.3%–3.0% 95% CI)
China [226,227]	General population	0.65% (0.66% men, 0.63% women)	General population	1.14% unadjusted. 0.71% age adjusted (0.72% men, 0.70% women) **34% newly detected AF**
England [228,229]	≥65 years	8.9% (7.9% to 9.7%) control; 8.4% (7.6% to 9.4%) opportunistic arm; 8.4% (7.6% to 9.3%) systematic arm.	≥45 years	2.0% over all (2.4% men; 1.6% in women) 8% ≥75 years. **≈29.5% newly detected AF**
Germany [230]				2.5% age weighted. 0.7% 35–44 years 10.6% 65–74 years. **15.5% newly detected AF**
Hong Kong [28]			General population	1.8% overall. (95% CI 1.6% to 2%) 2.8% men (95% CI 2.3% to 3.3%) 1.4%. women (95% CI 1.2% to 1.6%). **42.2% newly detected AF**
India [25,26,27]	General population	0.1–0.5%	General population	1.6% 5.6% (for ≥75years.)
Italy [231,232]	≥65 years	7.4%	≥65 years	7.3% overall (95% CI 6.6–8.1) 8.6% men. (95% CI 7.5–9.8) 6.2% women (95% CI 5.3–7.2) 16.7% >85years. **8.1% 2016 population adjustment** (95% CI 5.9–11.1)
Netherlands [9]	≥55 years	5.5% overall (0.7% for 55–59 years; 17.8% ≥85years)		
Portugal [233,234]	≥40 years	2.5% over all (2.2–2.8%: 95% CI) 6.6% (70 –79 years) 10.4% (≥80years)	≥65 years	9% overall (8.9% men; 9.1% women) **35.9% newly detected**
Spain [235,236]	25–74 years	0.7% 1.1% men. 0.3% women	≥40 years	4.4% (3.8–5.1 95% CI) 4.4% men (3.6–5.2 95% CI) 4.5% women (3.6–5.3 95% CI) 17.7% ≥80 years (14.1–21.3 95% CI) **10% newly detected AF**
Sweden [237,238]	General population	2.5% overall 2.8% in men 2.1% women 3.9 ≥35 years 6.3% ≥50 years 13.8% ≥80 years	75/76-year-old	14.3%. (95% CI 12.1–16.8) **5.2% newly detected AF** (3.8–7.7 95% CI)
Ghana [239]	Rural		≥50 years	0.3% overall (95% CI 0.1–1.0)
Tanzania [240]	Rural		≥70 years	0.67% overall (95% CI 0.33–1.01) 0.96% women (95% CI 0.42 – 1.49 0.31% men (95% CI 0.04 – 1.24)
Ethiopia [241]	Urban		≥40 years	4.3% overall

Legend: * Publication date may be somewhat later than date of cohort data collection.

AF burden is lowest in sub-Saharan Africa [29], possibly due in part to under-detection and non-recognition. However, it is associated with increased risk of stroke and heart failure [3,30] and recently, an increase in disability-adjusted life years (DALYs) from AF [31]. African Americans, however, have a lower AF risk than other US ethnic groups despite a higher burden of AF risk factors [21,32]. There are few population screening studies for AF across the whole African continent, and these have reported relatively low AF burden, coupled with a very significant stroke and heart failure burden, but large-scale screening is lacking in this region, representing an important knowledge gap [30,33]. Similarly, comparative data are somewhat limited for South and Central America [34,35,36,37]. In one 2020 publication with 153,152 participants from 20 countries aged 35–70 (mean 52), using population sampling between 2004–2012, there was a 12-fold variation in prevalence between regions, highest in North America, Europe, then China and South-East Asia, lower in South America, and lowest in the Middle East, Africa and South Asia [38]. There was also a continuous gradient of AF prevalence from low- to middle- and high-income countries. Notably, the AF-related stroke risk did not differ between regions based on income, but anticoagulation rate was low in LMICs and remains lower than in HICs [39], which may in part explain the similarity in AF-related stroke risk.

Additionally, AF occurs at a relatively younger age in some LMICs [40,41,42,43], though this may be explained by more rheumatic AF or an effect from lower general survival in LMICs. However, an integrated oral anticoagulant (OAC) program and service is likely to reduce the burden of AF-related strokes in the young cost-effectively [6]. AF is associated with increased risk of morbidity (up to five-fold increase in stroke, and lesser increases in heart failure, and cognitive impairment) and two-fold and 1.5-fold increase in all-cause mortality in women and men, respectively [44,45,46,47]. AF management imposes huge financial burdens on health systems with an estimated annual direct cost of $26 billion in the USA and AU$874 million in Australia [48,49]. The financial impact of AF management is most likely worse in LMICs with fragile health systems, but data are scarce [29]. The embolic complications and cost of AF-related stroke care may be mitigated by early diagnosis using systematic or opportunistic screening and effective anticoagulation for patients with actionable (unrecognized or undertreated) AF [50].

## 2. Primordial and Primary AF Prevention

Prevention of AF is of paramount importance given its rising incidence, associated disease burden, and cost. As a condition closely associated with ageing and co-morbidity, AF also demonstrates distinct race and sex trends not yet fully understood [51]. There is, however, a well-recognised constellation of underlying risk factors including obesity, diabetes, and hypertension pre-disposing to cardiovascular disease generally and AF in particular [52,53,54]. The main risk factors for incident AF are summarized in Table 2. Although robust data comparing underlying risk factors for AF between different regions of the world are sparse, some studies showed a relatively lower prevalence of older age, diabetes, and hypertension among African AF patients, though hypertension, heart failure, and valvular heart disease remain major risk factors for AF in sub-Saharan Africa [29]. Observational studies suggested links between AF incidence and markers of social deprivation, including lower socioeconomic status (SES), lower educational attainment, and single partnership status, while prevalence data are variable, most likely from lower AF screening and diagnosis in populations with social deprivation [55]. While higher cardiovascular risk factor burden in individuals with social deprivation may drive AF risk, the association is not fully explained. Lower formal education, lower neighborhood SES, and unmarried patients with AF have a higher risk of AF-related complications such as stroke, myocardial infarction, heart failure, and mortality [56,57].

**Table 2:** Main risk factors for incident AF.


**Demographic and socioeconomic factors** [242,243,244,245,246,247,248]	Age, male sex, Caucasian ethnicity, lower socioeconomic status and social deprivation, family history of AF
**Lifestyle** [242,243,244,249,250,251]	Smoking/tobacco use, alcohol intake, sedentary lifestyle, or vigorous exercise
**Cardiovascular conditions** [51,242,243,244,252,253,254,255,256,257]	Heart failure, coronary artery disease, vascular disease, rheumatic heart disease and valvular disease, congenital heart disease, heart rhythm disorders
**Health factors and other risk factors** [242,243,244,258,259,260,261,262,263]	Hypertension, dyslipidemia, diabetes mellitus, renal dysfunction, obesity, sleep-disordered breathing, chronic obstructive pulmonary disease, inflammatory diseases, surgery


Successful primordial and primary prevention requires a sustained long-term effort. Effective AF prevention will require primordial prevention of modifiable predisposing risks such as diabetes and rheumatic heart disease. Lifestyle modifications including weight loss to reduce obesity [58,59,60], no or moderate alcohol intake [56,57,58], as well as regular physical activity [61,62,63,64] may reduce the risk for developing AF. In addition, detection and diagnosis of sleeping disorders [65,66] and prevention of rheumatic heart disease by improved sanitation and housing as well as prompt treatment of streptococcal infections, may also effectively reduce global AF burden. While all this is well-recognised, there is limited clinical trial evidence to support specific primary prevention interventions for AF. EUROASPIRE V confirms that control of blood pressure, lipids, and diabetes among patients with high risk for cardiovascular disease remains poor [67], suggesting that current strategies for primary prevention of AF and cardiovascular disease are insufficient.

**Figure 1:**
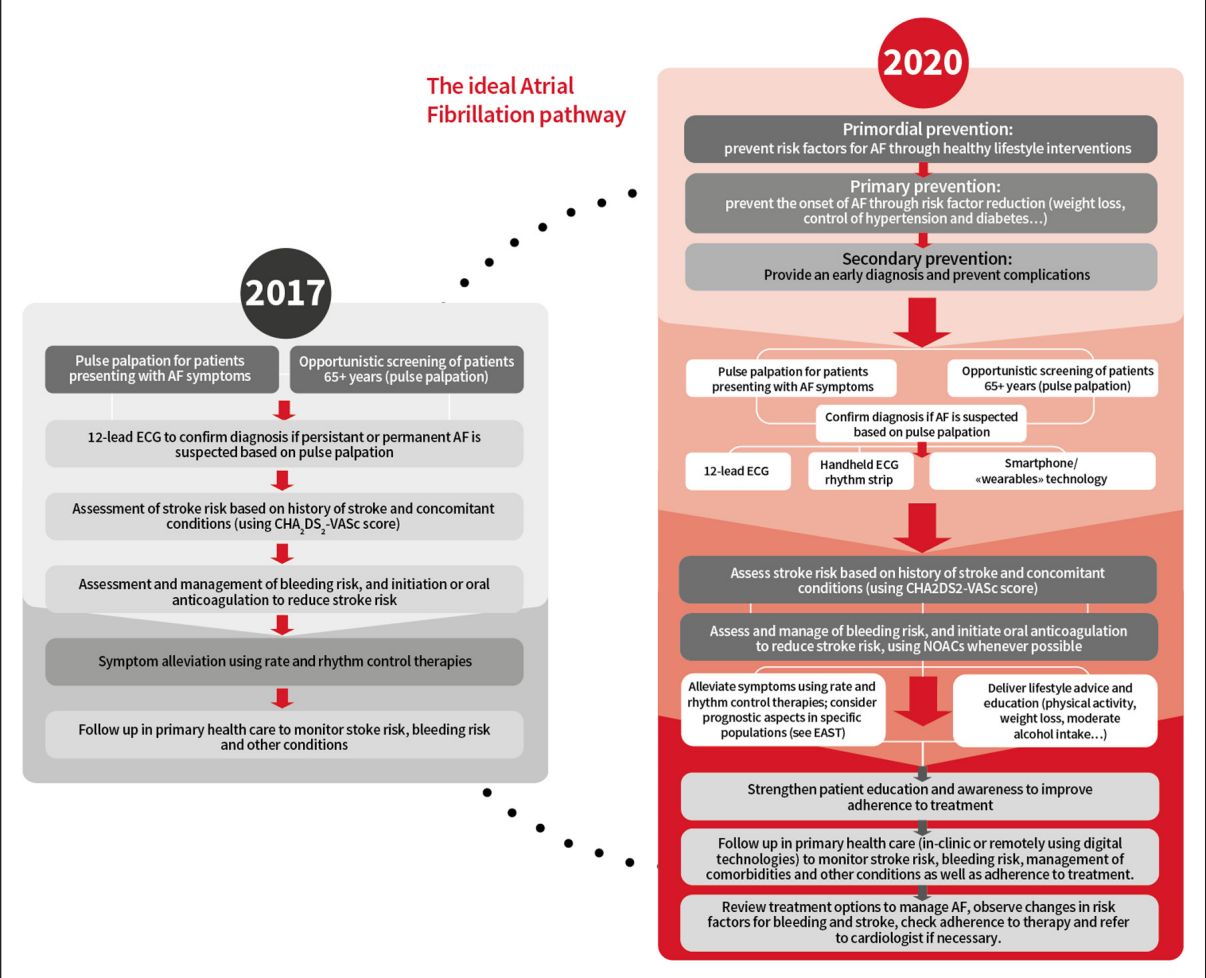
**Ideal AF pathway** © World Heart Federation.

For optimal benefit, individuals should actively participate in shared decision making and a team-based approach to deliver care, which promotes self-management and provides adequate support [5,68,69]. The use of individualized risk-assessment models and structured approaches to communication, such as motivational interviewing, could help to engage patients more effectively and enhance outcomes [70]. To achieve this globally is an ongoing challenge across all cardiovascular diseases with many shared risk factors and is the first part of our revised ideal AF pathway (Figure 1, Table 2).

## 3. Identifying Roadblocks and Strategies to Overcome Them

The following sections focus on a series of new opportunities to overcome roadblocks in the detection and management of AF. An updated overview of roadblocks and possible solutions based on the 2017 WHF Roadmap, WHF members’ feedback, and a review of the literature by the expert writing group is shown in Table 3.

**Table 3:** Roadblocks, strategies, and potential solutions.

Dimension	Roadblock	Strategy	Potential solutions

**Geographic accessibility**	Long distances to clinics result in low numbers of rural patients presenting to clinics for screening and follow-up appointments.	Improve accessibility of screening for rural populations. Strengthen capacity for ECG testing in remote areas. Promote the use of digital technology to improve screening and diagnosis of AF.	Train community health workers or pharmacists to screen for possible AF with pulse-checking in non-clinic settings. Educate in schools about checking pulse and relationship of AF with stroke. Educate at-risk populations (e.g., those 65+ years of age) to self-screen with pulse checks. Implement novel telemedicine technologies (e.g., transmission of ECG results from rural areas to urban facilities) including handheld digital rhythm strips (accepted by ESC for AF diagnosis). Use digital technology or ‘wearables’ to conduct non-invasive screening (e.g., PPG readings generated from smartphones, though ECG still required for diagnosis). Use digital technology for remote patient follow up (e.g., phone or video calls).
**Availability**	Shortage of health care professionals with training in AF, including interpretation of ECG, initiation of and monitoring of anticoagulation therapy. Absence of rhythm-control strategies Lack of integration of AF management services with other cardiology and medical care.	Raise awareness of AF among health care professionals. Reduce dependence on highly trained medical staff for AF screening and management. Implement coherent rhythm control strategies. Better integration with other cardiology and medical services.	Conduct awareness campaigns through healthcare professional networks. Improve postgraduate training and CME. Develop simple and locally applicable AF guidelines. Implement non-physician healthcare workers (NPHW)-managed anticoagulation program. Increase governmental funding. Progress towards Universal Health Coverage (UHC). Train human resources. Set up AF research and registries in LMICs to ascertain the disease patterns specific to these countries. Involve allied health professionals for monitoring and follow-up purposes. Rely on electronic solutions (e.g., smartphones and apps) to provide patients with regular guidance. Promote awareness of AF management in related medical services (hypertension, heart failure, coronary artery disease, medical). Treat and prevent contributory factors (e.g., hypertension, heart failure, coronary artery disease).
**Affordability**	OACs potentially unaffordable for patient households, resulting in nonadherence to treatment regime. Pharmaceutical poverty. Access to non-pharmacological rhythm control strategies, i.e., catheter ablation, LAAO.	Improve affordability of OACs and other essential medicines so that every patient can access them. Design novel treatment environments such as office-based labs.	Provide universal health care coverage for essential medicines, or provide similar support via a not-for-profit organisation). Implement internationally recognized policies for the reduction of essential medicine costs. Ensure that national essential medicines lists include NOACs. Promote the availability of NOACs as generics. Office-based labs provide safe and affordable spaces for interventions in AF patients.
**Acceptability**	Reluctance of physicians and patients to initiate anticoagulation therapy. Lack of awareness of importance of persistent adherence to OAC therapy.	Improve awareness of and capacity for managing OAC. therapy among physicians. Improve patient understanding of importance of OAC therapy and capacity to adhere to therapy.	Conduct country-specific training on OAC therapy management and support programmes for non-cardiologist health care professionals with the support of professional patient organisations when available. Develop and implement country-specific patient education, health literacy, and support programmes for diagnosed AF patients on OAC therapy and foster the dissemination of existing resources across countries. Support the development of structured patient organisations. Foster patient-centred approaches to support medication adherence and effective lifestyle risk reduction. Foster patient self-management and adherence to medication through digital technology and connected devices. Conduct research into feasibility of self-monitoring programmes for patients on OAC therapy in LMICs.
**Quality**	Unavailability of standards or norms to ascertain the quality of certain new devices, services, and treatments. Lack of patient-reported outcomes. Lack of a clear definition of quality indicators and markers, including specificities per regions.	Implement robust mechanisms for the accreditation/certification of new devices, services, and treatments. Rely on a set of standardised patient report outcomes. Adopt a globally acceptable definition of quality indicators and markers.	Create a list of certified devices, apps, etc. Ensure that technology is supported by a clear pathway to treatment. Foster implementation research. Promote the use of a standard set of patient-reported outcomes among health practitioners (195). Use a common definition of quality indicators and markers.

## 4. Opportunities for New Digital Technologies to Overcome Barriers in Screening, Diagnosis, and Monitoring for AF

### a. Mobile technology

AF is often paroxysmal [71,72,73,74] and frequently asymptomatic [75,76,77], as noted when implanted devices are interrogated [72]. Therefore, detection that relies on reported symptoms, intermittent pulse checks, or 12-lead ECGs will miss a sizable number of cases. Given the substantial thrombo-embolic risk of asymptomatic and/or paroxysmal AF [74,78,79], long-term monitoring with ambulatory ECG recordings or implanted loop recorders has been increasingly deployed to improve detection, particularly in high-risk clinical settings such as after cryptogenic stroke [80,81]. Digital technology in smart phones or ‘wearables’; however, it might allow long-term, non-invasive screening to improve AF diagnosis in broad populations across the globe.

Multiple investigators [82,83,84] have evaluated the use of photoplethysmography (PPG), a low-cost transdermal optical technique available on most fitness trackers, smartwatches, and smartphones that detects blood volume changes in the microvascular tissue bed, indirectly measuring heart rate and regularity of rhythm [85]. A prospective evaluation [86] of a PPG-based smartwatch algorithm in hospitalized patients confirmed a sensitivity of 93.7% and specificity of 98.2% for AF detection, though performance in real-world settings as opposed to research study environments are significantly worse. In asymptomatic younger people (mean age 35 and 40), two very large prospective trials in China [84] and USA [87] screened 187,912 and 419,297 people using Huawei and Apple PPG-enabled devices respectively, detecting ‘suspected AF’ in 424, and 2,161, but with confirmation of diagnosis in only a limited proportion through follow-up. While these studies suggest that the technology may develop into an effective tool, multiple barriers remain to be resolved: inadequate quality recordings in up to 22% of subjects, [84,86,88] lower reliability of PPG at higher heart rates, [85,88] cost/affordability for LMICs, and lower penetration of digital technology in older populations amongst whom AF is more prevalent [88,89]. Other technologies that support early AF diagnosis also rely on detection of regularity of the pulse wave using oscillometry in blood pressure sphygmomanometry [90] and even phasic changes in facial colour using a video camera [91]. Sphygmomanometers have been used in LMICs, but all of these pulse-based technologies suffer from the problem of requiring an ECG to make the diagnosis, which leads to delays or loss of follow-up through requirement for distant referral, as in a recent community study in Thailand [92]. In the end, a 12-lead ECG or 30-second ECG rhythm strip is required to make the diagnosis of AF [73].

Proprietary technologies already allow direct recording and interpretation of an ECG tracing from a smartwatch [93]. For example, the Kardia Band used an Apple smartwatch to detect possible AF from intermittent PPG registrations, with user wrist haptic/buzz notification to record an ECG from the band, followed by an automated ECG interpretation [94], but this device is no longer available. The same principle is now inbuilt in the Apple smartwatch (but not enabled in all countries outside USA) and is being used in the large prospective Heartline screening study (NCT04276441). Many more of these smartwatch PPG/ECG systems are now available in HICs, though not yet in most LMICs. While promising, the cost and technical issues listed above could limit global generalisability as scalable screening tools.

A number of stand-alone handheld or chest-applied ECG devices, or small inexpensive devices attached to a smartphone, are available and commonly used as event recorders providing an ECG rhythm strip for AF diagnosis when symptoms are suggestive or to estimate AF burden in known AF. These devices can also be used to screen for AF by either health professionals or personnel with minimal training in LMICs [25,95] or by individuals themselves [96,97], with single or multiple intermittent ECG 30-second snapshots [50]. As a 30-second rhythm strip is considered sufficient for AF diagnosis, this may be the preferred technology for making an AF diagnosis after non-ECG devices or pulse-based screening.

Although adoption of smart watch technology in LMICs is very low, surveys conducted in 11 LMICs in four global regions found that 53% of adults have access to a smartphone capable of accessing the internet and running apps [98]. Data from UNESCO suggests that one third of adults in developing countries already use smart phones, and the Global System for Mobile Communications (GMSA) predicts that largest growth in smartphone use by 2025 will be in sub-Saharan Africa [99]. Prospective data of self-recording fingertip PPG intermittently from a standard smartphone shows promise for detecting AF [83]. However, a 2020 survey of 588 healthcare professionals showed almost 70% believe we are not yet ready for mass consumer-initiated AF screening using wearables/apps [100]. This suggests that smartphones/PPGs may allow more effective global screening and diagnosis of AF at some stage in the future (possibly within the next decade), provided that the technology matures adequately, the burden of data analysis and follow-up can be managed, and issues of privacy are adequately anticipated.

### b. Artificial intelligence (AI) to predict AF

A potential alternative or addition to using wearables/apps and devices utilizes modern computing technology to examine existing clinical specimens, samples, or data (including ECG data) to identify patients at high risk of actionable AF, or even cardiovascular disease [101]. Such technologies could provide a means to identify patients at high risk of impending or undiagnosed paroxysmal AF or AF-related stroke, and thus might serve to identify those who could benefit from AF screening or potentially even anticoagulation, such as in the setting of stroke of uncertain source (ESUS) [81]. These technologies may also be essential to facilitating interpretation and integration of the massive amount of data collected using mobile and consumer-centered technologies.

A recent study showed that an AI-ECG algorithm trained using over 500,000 normal sinus rhythm ECGs from over 180,000 patients, had favorable performance characteristics for the identification of concomitant paroxysmal AF (area under the receiver operator curve [AUC] of 0.87, sensitivity of 79%, specificity of 79.5%) [102]. While still investigational and requiring validation in additional prospective cohorts that do not include cardioversion, the promise of this technology is as a low-cost and easily scalable intervention to identify patients with increased likelihood of paroxysmal or future AF from a single 12-lead ECG in sinus rhythm. Clinicians could even apply this technology retrospectively to existing ECG databases to segment a population by levels of risk for more intensive screening. A provocative case study recently described a patient with recurrent cryptogenic stroke in whom the AI-ECG identified a signal of AF risk many years before the onset of clinically recognized AF [103]. While studies examining empiric anticoagulation in patients with ESUS have not demonstrated a benefit of anticoagulation in the absence of documented AF [104,105], perhaps an AI-ECG could be used to identify patients in whom the risk-benefit balance would favor earlier anticoagulation.

Before such approaches can be implemented, considerable work is needed to externally validate these algorithms in diverse populations [106] and to test them prospectively with other monitoring approaches (clinicaltrials.gov NCT04208971). Furthermore, the lack of ‘explainability’ of current convolutional neural networks leaves many clinicians reluctant to accept the predictions that emerge from a ‘black box’. While the potential implications of AI-ECG in AF screening and treatment is only beginning to emerge, it is clear that significant questions remain.

### c. Wearable or implantable ECG monitors

Wearable ECG monitors are noninvasive, easy to use, and readily available compared to other medical devices [107] but may be relatively expensive for scaled use in LMICs. Currently, wearable continuous ECG monitors built into a belt, vest, or adhesive patch are available. Many are designed to measure heart or pulse rate for fitness rather than rhythm and are not appropriate for medical use. Some products, particularly adhesive patch ECG recorders (some waterproof allowing showering), have the capability to record and store continuous ECG recordings for up to two weeks, and have regulatory approval for detecting rhythms such as AF. The mSToPS trial revealed that use of a home-based self-applied 14-day patch ECG monitor improved AF diagnosis [108]. A wearable dry-electrode belt monitor worn around the chest for 30 days significantly improved AF detection in cryptogenic stroke [109]. There are a number of other wearable external devices and patches that permit continuous telemetry, but availability is variable. Implantable loop recorders have much higher detection rates of undiagnosed AF than these noninvasive ECG monitors [110] but at present are too expensive for AF screening at scale. A recent study simulating various AF screenings using implantable loop recorder as gold standard demonstrated that intermittent AF screening using three 24-hour monitoring periods are superior to one 72-hour monitoring: even with 30-day monitoring, 40% of AF will go undetected [111]. However, the AF missed by two-week intermittent recordings compared to continuous recordings has a lower AF burden, which may have lesser prognostic significance.

### d. Overcoming roadblocks

**Figure 2:**
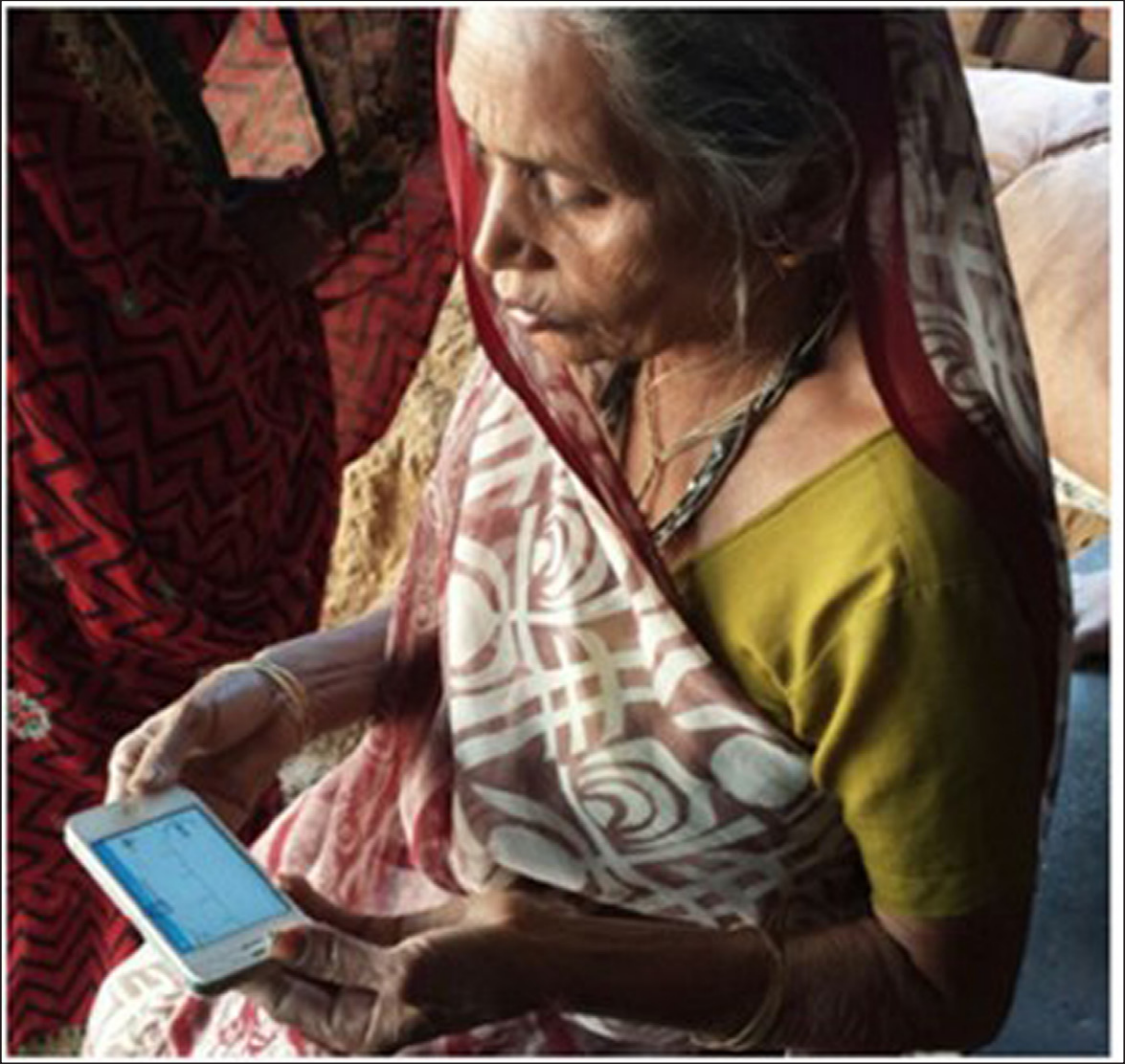
**Recording of ECG rhythm strip by a woman instructed by a village health worker using a mobile hand-held smartphone ECG device.** Reprinted from International Journal of Cardiology, 280, Soni A, Karna S, Fahey N, Sanghai S, Patel H, Raithatha S, et al., Age-and-sex Stratified Prevalence of Atrial Fibrillation in Rural Western India: Results of SMARTIndia, a population-based screening study, pp. 84–88, 2019, with permission from Elsevier.

Developments in technology certainly show promise for increasing detection and diagnosis of AF that may lead to stroke and other complications, which could be prevented by interventions such as OAC therapy. PPG on smartphones seems the most scalable option, but the diagnosis still requires an ECG for confirmation – that is, another test – which poses challenges. Smartphone ECG devices can provide an AF diagnosis without another test and have been used successfully for screening in many countries, including LMICs [25,28,112,1132,114] (Figure 2). However, many LMICs do not have a ready access to expert ECG readers required to confirm the rhythm, or even 12-lead digital ECG recorders with a valid automated algorithm. This might be facilitated by a global or country-specific ECG consultative service for digitally acquired ECGs as in Brazil [115]. We must also keep in sight that pulse palpation has sufficient sensitivity and specificity to be still recommended as a screening tool. It is readily available and certainly scalable in all countries: skills can be acquired by training health workers or by raising public awareness through organisations such as the Atrial Fibrillation Association (AFA). Whichever approach to screening is taken, there must be a system-wide mechanism in place to link diagnosis of new or actionable AF with an evidence-based management pathway including OAC thrombo-prophylaxis to prevent complications, and rate and rhythm control where required.

## 5. Rate-Control and Rhythm-Control Strategies – Perspective and Challenges

### a. Rate-control vs. rhythm-control strategy

The decision for rate or rhythm control strategy in patients with AF is one of the key elements in the patient treatment pathway. Major AF guidelines, including the new 2020 ESC guideline, have defined the patient’s symptoms as leading criterion for rate- or rhythm-control therapy selection [73]. This recommendation is based on a number of clinical trials showing no difference in major outcome measures including stroke and mortality between these treatment strategies [116]. Although one very recent clinical trial comparing early rhythm-control intervention with usual care (rate-control) suggests advantages for an early rhythm-control strategy (in a selected subgroup of ‘early’ AF) [117], the main decision pathway for the majority of AF patients will remain based on symptoms in the foreseeable future. Importantly, the indications for long-term anticoagulation to reduce risk of thromboembolism, particularly stroke, in patients with AF stroke risk factors, are identical for both treatment strategies [118,119].

### b. Methods for rate- or rhythm-control

Rate-control is achieved by pharmacologic slowing of atrio-ventricular conduction mainly with beta-blockers, calcium antagonists, and digitalis [120,121,122]. These drugs are usually well tolerated and globally available at relatively low cost [123]. Rate-control therapy can be applied in ambulatory settings, but may necessitate 24-hour ECG monitoring, not readily available in LMICs [124]. Rhythm-control is medically more complex as it may involve initial interventions such as pharmacological or electrical cardioversion that require a medical center/hospital setting and prescription of antiarrhythmic drugs for maintenance of sinus rhythm [125]. Lifestyle modifications, especially weight control, are also important for symptom control and reducing symptoms [126,127]. Compared to rate-control drugs, antiarrhythmic drugs for rhythm-control carry a higher risk of significant cardiac and extracardiac side effects so therapy requires closer medical monitoring and follow-up [125]. Moreover, as most membrane-active antiarrhythmic drugs are contraindicated in patients with structural heart disease (particularly ischemic heart disease and advanced heart failure), exclusion of such disease (including ECG, cardiac imaging, and in selected cases cardiac catheterization) is often necessary before therapy initiation [128,129,130,131,132,133,134]. Thus, in geographies without such diagnostic and monitoring facilities it is difficult and potentially risky to promote rhythm control with antiarrhythmic drugs. For selected patients with symptomatic AF, catheter ablation has evolved as an alternative rhythm-control strategy [135[136[137,138]: it is more effective than antiarrhythmic drugs and proven relatively safe [139]. Application of catheter ablation is highest in HICs in Europe and North America. It is also increasingly used in many Asian countries and South America [140,141], but is available in only seven African countries (Algeria, Egypt, Kenya, Libya, Morocco, South Africa, Tunisia) [142,143], mostly with low caseloads. In general, catheter ablation rates will be higher in the private than public health sectors, and this differential may be greater in LMICs.

### c. Challenges and roadblocks for rhythm-control strategies in LMICs

The field of arrhythmia management has evolved in a disconnected way across LMICs with significant variation in local expertise, cost, and uptake of management strategies. Recent surveys across LMICs found wide and glaring gaps in availability of and access to catheter ablation and antiarrhythmic drugs and services [142,144]. AF rhythm-control is generally rarely employed in many LMICs [145]. The same roadblocks highlighted in the original WHF Roadmap [1] as contributing factors to the care gaps in rate management and anticoagulation of patients with AF apply to rhythm management, with some additional ones. These numerous challenges include:

- *Limited antiarrhythmic drug availability:* Amiodarone is the only AF cardioverting agent available in >60% of the countries surveyed; other drugs are largely unavailable. No AF-cardioverting antiarrhythmic drugs are on the essential medicines lists of surveyed countries [142,144].
- *Workforce shortage:* There are few trained cardiologists and electrophysiology physicians in many LMICs. Interventional treatment of complex arrhythmias, such as AF ablation, requires highly skilled personnel. In the PASCAR surveys, 15% of countries in Africa did not have a single general cardiologist let alone a specialist electrophysiologist. There is a critical lack of specialty training programs in LMICs [142,144].
- *Lack of specialized facilities:* including cardiac catheterization laboratories and electrophysiology equipment. For example, 55% of African countries have no electrophysiology laboratory; only six countries reported cardiac catheterization laboratories with 3D mapping systems required for complex ablation procedures like AF.
- *Lack of political will:* Many LMIC governments do not see establishment and support of hospitals, specialist training, and specialized EP equipment purchase and maintenance as a priority [142,144].
- *High cost* of AF interventional infrastructure relative to the overall public health budgets [1].
- *Low level of clinical research and international collaboration* [146].
- *No local AF clinical practice guidelines or prospective registries*.
- *Lack of or inadequate public health policies or healthcare system:* Few LMICs provide universal health coverage to their populations [147,148,149], resulting in heterogeneous access to healthcare infrastructure. Public health orientation and patient education may not be priorities and will adversely affect adherence to rhythm-control drugs.

## 6. Interventional Prevention of Cardioembolism by Left Atrial Appendage Occlusion (LAAO)

Percutaneous LAAO and surgical LAAO/exclusion are non-pharmacologic strategies for stroke prevention [150,151,152], as the LAA is the main source of cardiac thrombi leading to arterial embolization and stroke. In two relatively small clinical trials LAAO has been shown to be non-inferior to OAC with warfarin for a composite endpoint including stroke [153]. Multiple registries and observational studies indicated safety and efficacy for stroke prevention; however, efficacy compared to NOAC therapy (current standard of care for stroke prevention) has not yet been evaluated in a randomized trial. The most frequent accepted indications for LAAO are high stroke risk with contraindications to NOAC/OAC treatment (mainly severe bleeding risk/complications) or AF-related stroke despite effective anticoagulation, but LAAO needs further validation before it can be widely recommended. In experienced hands LAAO is effective and relatively safe [154], though serious complications may be more common in practice than in trials, and device thrombosis may not be benign [127]. Additionally, the same human and equipment resource limitations as for catheter ablation, including onsite cardio-thoracic surgical back-up for complications, largely limit LAAO to HICs. LAAO is unlikely to play a major role for stroke prevention in LMICs using a 10-year perspective.

## 7. Anticoagulant Therapy to Prevent AF-Related Stroke

Appropriate initiation of anticoagulant therapy is the key intervention for AF-related stroke reduction in patients with stroke risk factors [127]. Benefits of oral anticoagulation with VKA and NOACs have been clearly demonstrated in multiple large-scale trials. However, NOACs have distinct advantages over VKA as they do not require monitoring and have fewer drug and diet interactions. In addition, they reduce stroke, intracranial hemorrhage, and mortality [155]. Thus, recent guidelines clearly recommend preferred use of NOACs over VKA [127].

### a. Addition of NOACs to WHO Essential Medicines List (EML)

Despite guideline recommendations, NOACs are infrequently used in low-resource settings due to barriers of cost and accessibility. In 1977 the WHO EML was established to address inaccessibility of many medications to certain populations [156]. Medicines added to the EML must be available in the correct dosage, with quality assurance and in adequate amounts for population needs and should reflect population health priorities [156]. They are intended to guide development of national EMLs adopted in national policy and practice.

In 2019, the EML panel reviewed efficacy, safety, and cost-effectiveness of NOACs following a submission by members of the WHF Emerging Leaders Programme, concluding that current evidence supported global use. Consequently, NOACs were added to the WHO EML, an important first step in global prevention of AF-related stroke, especially in LMICs, where OAC therapy uptake is low. The next step requires advocacy to ensure national EMLs include NOACs, other steps to increase affordability [157] followed by action to include implementation into local policy and practice.

### b. Implementation of OAC therapy for non-valvular and valvular AF in HICs and LMICs

In HICs, OAC use seems to be increasing in the past 10 years. For example, between 2006 to 2016, OAC use in the UK increased from <50% to almost 80%, mostly in the latter five years after NOACs were available [158], and similar HIC increases have been shown in the Garfield Registry [39]. A more than two-fold increase (to 35.6% in 2015) in OAC prescription rates (73% NOACs) was observed in Taiwan [159], while in Korea, OAC use rose from 34.7% to 50.6% during 2008–2015 (almost half NOACs) [160]. Modelling indicated that increasing OAC use was associated with declining stroke rates in both UK and Taiwan [158,159], a strong indicator of the value of achieving a global increase in OAC uptake for AF.

Despite these improvements, OAC use is not consistent at a global, country, or even regional levels. Many LMICs lack data on AF management, even though the population in these countries may be at higher AF-related stroke risk. Contemporary registry studies and trials [39,161] from various geographical regions have consistently shown widespread but extremely variable OAC underuse for AF, more severe for LMICs [29,40,41,42,43,162]. Prevalence of stroke among AF patients in LMICs range from 10.7% to 27% [29], compared with 5% in HICs [41]. OAC use was as low as 2.2% in patients with AF and a history of stroke in China, according to the community-based National Stroke Screening Survey conducted during 2013–2014 [163]. In contrast, OAC use increased to 36.5% during the same time period among hospital-based patients in the capital city of Beijing [164]. These differences highlighted that OAC use may be influenced by factors such as physician specialization, patient medical literacy, and population subgroup economic status.

AF and stroke related to rheumatic valvular heart disease (RVHD) remains a significant problem [27,43]. The current recommendation of VKA for AF with RVHD produces continuing need for international normalized ratio (INR) testing, which can be problematic. There is a need for research on use of NOACs versus VKA in RVHD [165], given their non-inferiority in bioprosthetic valves [166]. Inclusion of point-of-care INR testing in OAC programs would avoid travel costs [27,167,168]. This is also important for NVAF when NOACs are not available, as time in therapeutic range (TTR) is lower in LMICs even in clinical trials [169]. To maximize INR TTR and ensure drug adherence, incorporating smartphone-based apps guiding VKA dose will be helpful. Use of NOACs over VKA should be encouraged in all NVAF at risk of stroke. This could be implemented by use of generic NOACs and national government-funded programs to reduce stroke as previously outlined.

Even within HICs, pockets of social deprivation and poverty can produce disparities in OAC usage that may require a different approach. High AF risk at a younger age and low OAC utilization are seen in first nation peoples and minorities [170,171,172].

### c. Strategies to increase OAC uptake and promote adherence and persistence, such as physician education, patient education, and enhanced health literacy

Many of the strategies described in *i* to *vii* are equally applicable to risk-factor management and rate and rhythm control.

#### i. Issues for LMICs

**Figure 3:**
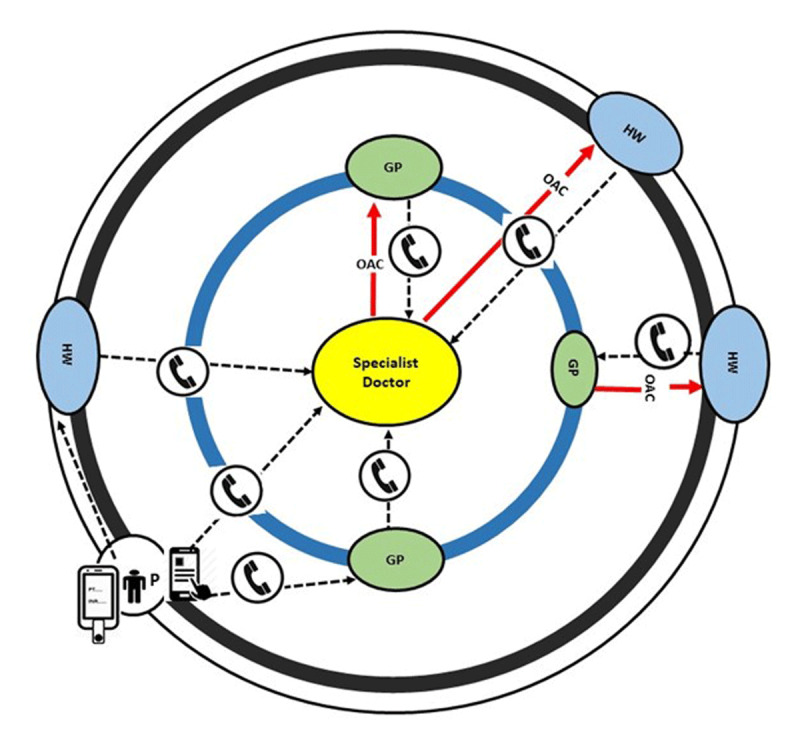
**Proposed hub-and-spoke model of oral anticoagulant therapy in patients with atrial fibrillation in low- and middle-income countries.** Specialist doctor at hub – If no specialist is available, the hub may be a GP. GP – general practitioner, HW – health worker at spoke. P – the depicted Patient (P) here has point-of-care INR monitoring facility and dosage adjustment and data sharing app.

To improve OAC use in LMICs calls for a team approach. Delivery of anticoagulation services can be expanded to a large geographical area through a hub-and-spoke model (Figure 3), applicable particularly for rural and semi-urban areas. In this model, a specialist physician trained in anticoagulation therapy serves as the expert at the hub, while general practitioners (GP), and community health workers, including trained nurses, operate the spokes at two levels. Case detection and patient follow-up can be carried out at the periphery by health workers, while initiation of OAC therapy, patient evaluation, and complex issues around anticoagulation or choice or rhythm- or rate-control strategy are carried out in consultation between the GPs and the specialist physician (in person or by tele-consultation). Standard anticoagulation services including point-of-care INR testing can be carried out in the periphery close to the patient. Setting-specific apps can facilitate OAC and other management, but patient involvement with apps will depend on educational and economic status. Workflow can be integrated through telemedicine, which acts as the bridge between spoke and the hub.

LMICs face a number of challenges in providing cost-effective antithrombotic therapy in AF patients. Monitoring quality of anticoagulation for VKA to achieve the minimum TTR threshold is required for optimal results [161]. Robust cost-effectiveness analyses, taking into account short-term budget impact and longer-term economic impact of strokes prevented, are important for LMIC decision makers to consider switching from VKA to NOACs: in Brazil NOAC use may even lower total healthcare costs [173]. Anticoagulation for AF is suboptimal in LMICs: improving physician awareness is crucial, as nearly half of patients not receiving OACs result from physician choice [39]. Periodic healthcare provider education and 24/7 hub backup helpline will ensure accessibility of safe and effective OAC therapy. Hub back-up for ECG rhythm diagnosis is another potential advantage of this model.

#### ii. Medication adherence

Medication adherence to durable anticoagulation therapy is essential for reducing AF-related stroke and thrombotic events, health-care related cost, and rate of hospitalizations [174]. Although long-term treatment with OACs is emphasized in different guidelines, both adherence and persistence with treatment is suboptimal and variable in real-world studies from 23% to 53–95%) [174,175,176,177,178,179,180,181]. Multiple possible factors are associated with higher medication adherence: female gender, older age, lower body weight, anticoagulation-naïve user, lower medication dose, and electronic transmission of prescriptions [177,180,182,183,184,185]. Non-adherence increases with polypharmacy, particularly pertinent in AF patients typically on five medications daily [186]. Some non-adherence is unintentional and relates to forgetfulness: memory aids and establishing routines can improve adherence, particularly for the elderly or cognitively impaired. Health care providers managing AF patients should not only estimate the risk of non-adherence, but also provide solutions including improving patient’s knowledge or using shared decision-making tools to increase medication adherence and persistence [187,188]. Some medication non-adherence, particularly non-persistence, is intentional and influenced by beliefs about medications, a component of health literacy. OAC non-persistence is associated with a continuing risk of avoidable stroke [189,190].

#### iii. Health literacy

Health literacy refers to the appropriate skills and knowledge needed to understand, and the confidence to access, evaluate, utilise, and navigate health care [191]. It has been demonstrated that higher levels of AF-related knowledge not only improve therapy uptake and adherence but also reduce AF-related complications such as stroke, thrombotic events, as well as health-care related costs and rate of hospitalizations [73].

Two multinational surveys showed that more than half of the patients with AF had low levels of knowledge about the increased risk of stroke related to AF or did not even know the name of their condition [192,193]. A recent European Heart Rhythm Association (EHRA) survey showed there is still room for improvement in education of patients taking OACs, mainly for NOACs [194]. Therefore tailored, context-specific strategies to increase medication adherence and persistence are essential.

#### iv. Patient knowledge gaps

Identification of deficiencies of patients’ knowledge plays a substantial role in designing and conducting evaluation of effective patient education platforms [195,196]. The following items have been identified as the most common knowledge gaps: recognizing AF presentations, AF risk factors, awareness about their diagnosis, drug and food interactions of OACs, dietary vitamin K food content and intake, meaning of INR and its interpretation, how to handle missed OAC doses, and OAC side effects [197]. Little or no data about patient knowledge of rate and rhythm medications are available. Patient support groups like the AF Association and Arrythmia Alliance could play an important role both in assessing knowledge in different countries or regions and in filling the gaps.

#### v. Educational interventions

Educational intervention programs to improve patient knowledge, particularly about therapy, could significantly impact therapeutic adherence, persistence, and therefore efficacy [198]. An easy to use online tailored-education platform improved AF- and procedure-related knowledge in patients, with a durable effect for at least 12 weeks post-ablation [199]. Another simple educational intervention improved therapeutic adherence 15–25%, and reduced stroke [200,201]. More recently the IMPACT-AF study, including education of patients, their families, and health care providers, led to a significant increase in OAC treatment and significantly fewer strokes. This suggests that education needs to be extended to health care professionals and patient families to achieve increased prescription and appropriate advice for patients [201]. In another trial, 3324 patients with AF were randomized to receive usual care or integrated care with a mobile AF application (mAFA) [202]. The integrated care arm provided clinical support tools for doctors and an educational programme combined with dynamic risk monitoring. The composite outcome of ‘ischemic stroke/systemic thromboembolism, death, and rehospitalization’ was significantly lower with mAFA compared to usual care [202]. Although the influence of educational interventions on quality of AF management are not definitive, studies highlight the importance of such interventions in increasing patients’ satisfaction with clinical decisions, establishing a therapeutic alliance with the care team to decrease decisional conflicts, and increase therapeutic adherence [201,203,204,205].

#### vi. Patient educational resources

**Figure 4:**
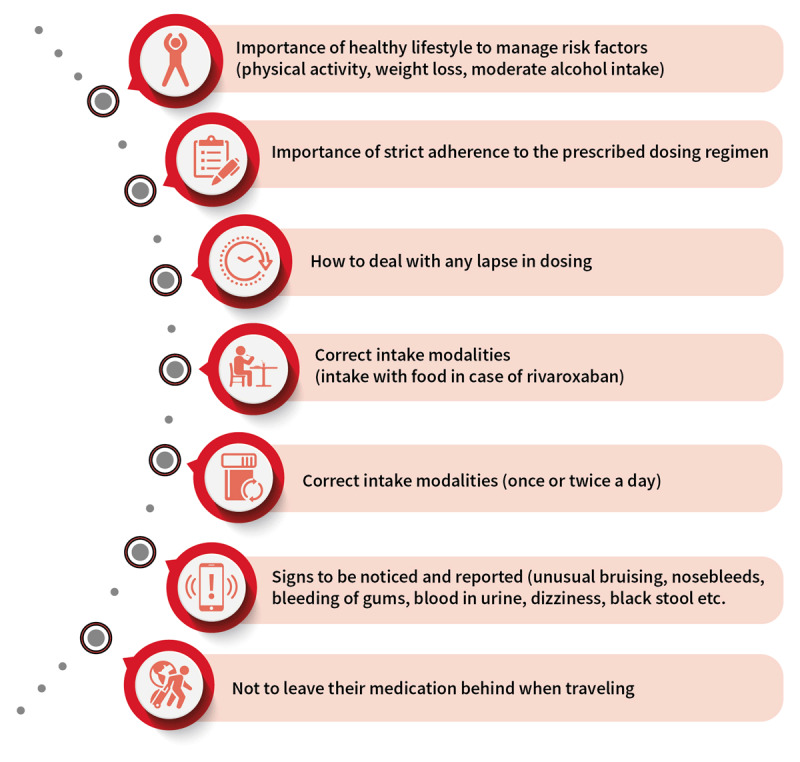
**Key educational points to convey to the patients with atrial fibrillation at each visit by physicians.** © World Heart Federation. Adapted based on the 2018 European Heart Rhythm Association Practical Guide recommendations [264].

Most physicians (80–95%) deliver patient information themselves, 30% recommend information brochures, and around 25% refer patients to other clinics specialized in OAC management for further education [206]. Only 9–15% of physicians refer patients to educational websites, a lost opportunity, while more than 60% of patients search for their disease and diagnostic or therapeutic-related information in the internet pages [199]. Among available AF patient education resources, the EHRA NOAC anticoagulation card is one of the most reliable, containing the critical educational issues in easy to understand points for patients to be delivered by healthcare practitioners (Table 4, Figure 4) [207]. This card is available for download in various languages at www.NOACforAF.eu. Patient-led organisations such as the Arrhythmia Alliance and the AF Association also provide valuable information, awareness, education, and support but are not active in many LMICs. Establishing such networks would be valuable.

**Table 4:** Educational items for anticoagulant medication adherence to be delivered by physicians or other health professionals to patients with atrial fibrillation.


**Important patient instructions**
A non-vitamin K antagonist anticoagulant (NOAC) thins the blood and reduces the risk of getting dangerous blood clots, in the same way as vitamin K antagonists (VKA, e.g., warfarin). Not taking the drug means no protection! Take your drug exactly as prescribed (once or twice daily for NOAC, once a day with the correct dose for VKA). Do not skip a prescribed dose to ensure optimal protection from blood clots and stroke! Do not stop your medication without consulting your physician.
For NOACs, you may need occasional creatinine blood tests to check kidney function
For VKA, ensure a stable diet of vitamin K containing foods, and have your INR checked regularly to make sure you have optimal anticoagulation protection against clots without increasing bleeding risk.
After a trauma or bleeding event, consult with your physician regarding further management Do not add any other medication without consulting your physician, not even short-term painkillers that you can get without a prescription. You may need INR testing after starting any new medication if you are taking VKA Alert your dentist, surgeon, or another physician before an intervention.
**What to do in certain occasions**
**When should I contact a healthcare provider?**
Bleeding is the most common side effect of an anticoagulant. However, the reduction in the risk for stroke outweighs the bleeding risk. Contact your healthcare provider if you have any signs or symptoms of bleeding such as:
Unusual bruising, nosebleeds, bleeding of gums, bleeding from cuts that take a long time to stop Menstrual flow or vaginal bleeding that is heavier than normal Blood in urine, red or black stools Coughing up blood or vomiting blood Dizziness, paleness, or weakness
**What should I do if I missed a dose of NOAC?**
You should still take that dose unless the time until your next dose is less than the time after your missed dose.
**What if I accidentally took two doses of NOAC?**
Twice daily NOAC: you can opt to forgo the next planned dose and restart after 24 h. Once daily NOAC: you can continue the normal regimen without skipping a dose.
**What if I missed a dose of VKA or accidentally took an extra dose?**
Continue your normal dosing if you missed a dose. Omit one dose and have an INR check if you took a double dose


* This table is adapted from the 2018 European Heart Rhythm Association Practical Guide on the use of non-vitamin K antagonist oral anticoagulants in patients with atrial fibrillation [264].

Technology effectively supports patient self-management and can facilitate medication adherence. Connected devices provide information about wider health and well-being issues related to AF, quite apart from ability to monitor for AF as previously discussed. It is essential that diagnostic technology is supported by a clear treatment pathway to ensure anxiety is not heightened by an abnormal result [208]. Paradoxically, patients who understand more about their AF may have greater anxiety than those who don’t, but are also more likely to adhere to guideline-based therapy [209]. Therefore, a careful balance is necessary to ensure people understand but are not distressed by potential risk. Mobile Health applications (mHealth) also have a substantial role in supporting patients to self-manage, both alone and in consultation with health care professionals (eg Figure 3). mHealth apps can provide updated, clinically relevant, and personalised information to individuals, offering far-reaching support for individuals, because they are capable of delivering targeted and tailored evidence-based educational content alongside tracking functions designed to enhance self-management.

#### vii. Role of nurse-based support, physician assistants, non-physician staff

In the context of shared decision, nursing and allied professionals have an important role to play in supporting patients through detection, management, and treatment of AF. Successful community AF screening programs (such as the Arrhythmia Alliance Know Your Pulse campaign) have been led across the globe by patient organisations, pharmacists, and nurses, with high levels of participant satisfaction. Management of AF requires a patient-centered approach to support medication adherence and effective lifestyle risk reduction. Guidelines now recommend an ‘integrated-care’ approach, involving primary care physician, exercise specialists, dietitians, pharmacists, specialist nurses, psychologists, podiatrists, sleep physicians, as well as electrophysiologists, and cardiologists [73,210], which could be implemented in the hub-and-spoke model as previously described (Figure 3). A systematic review and meta-analysis of integrated care in AF demonstrated reductions in all-cause mortality and cardiovascular hospitalisations.

Access to a range of specialists including professional patient organisations can support people with AF to self-manage. Management of AF requires a collaborative approach with the patient and family at the centre of all decisions [211]. In AF, the majority of patients will need to take lifelong medication, especially OAC, to reduce their risk of AF-related stroke. Unless the patient is highly symptomatic, medication is usually given for the purpose of risk reduction, such as antihypertensives or OACs. These medications treat largely ‘silent’ risks, have no short-term benefit for the patient, and may cause side effects – which makes adherence and persistence challenging. Furthermore, lifestyle risk reduction is important, as studies have shown that weight loss, being physically active, and reducing alcohol consumption reduce occurrence and symptoms of AF, and non-physician support can be vital [127]. Because AF is multi-factorial, a team approach is beneficial to address the complex nature of the condition. Use of the ABC pathway (Atrial fibrillation Better Care pathway, including A = avoid stroke, B = better symptom control, C = cardiovascular risk factors and comorbidities management [212]), as advocated in ESC guidelines [127], could assist team management of all aspects of AF. In low-resource or remote settings, access to a specialist cardiologist may not be possible, but other members of the interdisciplinary team may be able to facilitate specialist support through access to videoconferencing, decision support aids, and mobile app-based support (Figure 1, Figure 3).

## 8. Additional considerations: Role of AF in heart failure and dementia/cognitive decline

While stroke prevention has been the primary focus of therapy for AF, heart failure is the most common cause of death among individuals with atrial fibrillation, and one of the leading causes of hospitalization [213,214,215]. The risk factors for AF and heart failure vary across the world. Hypertension is prevalent in all regions, but older age, obesity, and ischemic heart disease are more common in HICs, while rheumatic and valvular heart disease are more prevalent in LMICs [43]. Many of these risk factors are possible targets for both primary prevention of AF and prevention of its complications, including stroke and heart failure [216]. There is also evidence that an increased burden of AF is associated with worsening of heart failure symptoms [217]. There is a need for greater focus on heart failure prevention strategies which might parallel the success of stroke prevention in AF. Although randomized trials of anti-arrhythmic drug therapy failed to show a reduction in cardiovascular outcomes [218], catheter ablation of AF shows promise for patients with AF and heart failure [219].

In addition to stroke, patients with AF are at increased risk of silent cerebral thromboembolic lesions [220,221], and of worsening cognitive function over time [222]. It has been hypothesized that AF may lead to cognitive decline via repeated, small cerebral thromboembolic events, and that these might be preventable by OACs. Research is ongoing to determine if NOACs can prevent cognitive decline in AF, including patients with only subclinical AF [223], and those with clinical AF but no additional stroke risk factors [224]. Given the global burden of cognitive impairment and the morbidity and costs associated with dementia, there is an urgent need to determine whether OAC will reduce progression of cognitive impairment, even in those without a current definite indication for OAC thromboprophylaxis for stroke.

## 9. Prioritization

The global impact of AF is evident from current epidemiological data and projections. Aside from the impact of AF on quality of life, the deleterious effects of AF on stroke and heart failure on healthcare costs including hospital admissions will only increase the economic burden of the condition. As a result of the COVID-19 pandemic, there has been an understandable concentration of the health care sector on this infectious disease. This has had an adverse and hopefully transient impact on non-committable diseases such as AF.

The focus of this Roadmap update was to identify what has changed in the recognition and management of AF, particularly the emerging role of mobile technology and mHealth, and to consider the advances described in recent publications, many summarized in the 2020 ESC guideline on AF [127]. Most of the identified barriers and priorities in the 2017 WHF Roadmap for AF are still valid in 2020, as evident from the survey of its members that the WHF conducted alongside this update. This update has therefore redefined the global priorities for AF management and emphasized those especially important for LMICs.

- **Advocacy:** Dissemination of knowledge on the importance of AF as one of the leading issues of cardiovascular disease must be improved. Too many decision makers in the health care sector do not know the threats of AF precisely and intensely enough. The campaign to change this should be led by the continental cardiac societies (AHA, ASC, ESC, ACC, HRS, EHRA, APHRS, SOLEACE, PASCAR, and others), national heart societies and foundations, patient organisations such as the Arrythmia Alliance and AF Association, and civil organisations, supported and united by the World Heart Federation.
- **Implementation:** There are still gaps in implementation of guidelines for management of AF, particularly in LMICs. Although patient education is important, improving healthcare provider awareness and education updates is crucial. Periodic educational courses and 24/7 hub backup helpline are examples of effective methods to increase adherence to guideline-based therapeutic approaches.
- **Access:** Aside from knowledge dissemination and implementation, global access to cost-effective health care resources must be achieved. Priorities are precise diagnosis and access to pharmacologic treatment, preferentially to modern oral anticoagulation using NOACs [157], and also to rate control medications.
- **Literacy:** Improving health literacy among patients and using shared decision-making tools by improving adherence to and long-term persistence with therapy will reduce AF-related complications and health-care related costs. Therefore, promotion of tailored, context-specific strategies to increase medication adherence and persistence should be considered as one of the priorities for all health-care units.
- **Integrated Care:** In the context of shared decision-making, use of an ‘integrated-care’ approach for AF management to further improve the structured management of patients, promote patient values, and finally improve patient outcomes should be prioritized in all countries, irrespective of income status.
- **Digital Transformation of Care:** Use of mHealth technologies to educate patients, provide patient support in areas with less health care facilities, increase adherence to lifestyle and pharmacological therapeutic approaches, and facilitate AF screening should be considered as one of the priorities in the next years. At the same time, finding practical solutions for the previously mentioned potential barriers for using these technologies, mainly for AF screening using wearables/apps, should be kept in focus. Moreover, digital technologies will enable the systemic implementation of quality indicators for several domains in the AF management pathway.
- **Prevention and Screening:** Much more attention should be paid to AF preventive strategies including development of new strategies. Both physicians and patients need to be better informed about the important role of risk factors and lifestyle in AF development and recurrence after a successful ablation or other rhythm control strategy. Identification of unrecognized AF using technological advances is another important priority for prevention of complications, especially stroke.
- **Overcoming Barriers:** Local stakeholders at the national level should hold roundtables to improve understanding of local barriers and develop practical solutions to identified local barriers, thus contextualizing and adapting the AF roadmaps. Publishing the result of such interventions is encouraged to increase understanding of remaining problems and to generate solutions from other experts.

## References

[1] Murphy A, Banerjee A, Breithardt G, Camm AJ, Commerford P, Freedman B, et al. The World Heart Federation Roadmap for Nonvalvular Atrial Fibrillation. Glob Heart. 2017; 12(4): 273–84. DOI: 10.1016/j.gheart.2017.01.015 28336387

[2] Zaidel EJ, Leng X, Adeoye AM, Hakim F, Karmacharya B, Katbeh A, et al. Inclusion in the World Health Organization Model List of Essential Medicines of Non-Vitamin K Anticoagulants for Treatment of Non-Valvular Atrial Fibrillation: A Step Towards Reducing the Burden of Cardiovascular Morbidity and Mortality. Glob Heart. 2020; 15(1): 52. DOI: 10.5334/gh.608 32923346 PMC7413134

[3] Chugh SS, Roth GA, Gillum RF, Mensah GA. Global Burden of Atrial Fibrillation in Developed and Developing Nations. Glob Heart. 2014; 9(1): 113–9. DOI: 10.1016/j.gheart.2014.01.004 25432121

[4] Wann LS, Alpert LS, Calkins H, Cigarroa JE, Cleveland JC, Jr., et al. AHA/ACC/HRS guideline for the management of patients with atrial fibrillation: A report of the American College of Cardiology/American Heart Association Task Force on Practice Guidelines and the Heart Rhythm Society. J Am Coll Cardiol. 2014; 64(21): e1–e76. 24685669 10.1016/j.jacc.2014.03.022

[5] Kirchhof P, Benussi S, Kotecha D, Ahlsson A, Atar D, Casadei B, et al. 2016 ESC Guidelines for the management of atrial fibrillation developed in collaboration with EACTS. Eur Heart J. 2016; 37(38): 2893–962. DOI: 10.1093/eurheartj/ehw210 27567408

[6] Chugh SS, Havmoeller R, Narayanan K, Singh D, Rienstra M, Benjamin EJ, et al. Worldwide Epidemiology of Atrial Fibrillation. Circulation. 2014; 129(8): 837–47. DOI: 10.1161/CIRCULATIONAHA.113.005119 24345399 PMC4151302

[7] Haim M, Hoshen M, Reges O, Rabi Y, Balicer R, Leibowitz M. Prospective National Study of the Prevalence, Incidence, Management and Outcome of a Large Contemporary Cohort of Patients With Incident Non-Valvular Atrial Fibrillation. J Am Heart Assoc. 2015; 4(1): e001486. DOI: 10.1161/JAHA.114.001486 25609415 PMC4330072

[8] Björck S, Palaszewski B, Friberg L, Bergfeldt L. Atrial Fibrillation, Stroke Risk, and Warfarin Therapy Revisited. Stroke. 2013; 44(11): 3103–8. DOI: 10.1161/STROKEAHA.113.002329 23982711

[9] Heeringa J, van der Kuip DAM, Hofman A, Kors JA, van Herpen G, Stricker BHC, et al. Prevalence, incidence and lifetime risk of atrial fibrillation: The Rotterdam study. Eur Heart J. 2006; 27(8): 949–53. DOI: 10.1093/eurheartj/ehi825 16527828

[10] Nielsen JC, Lin YJ, de Oliveira Figueiredo MJ, Sepehri Shamloo A, Alfie A, Boveda S, et al. European Heart Rhythm Association (EHRA)/Heart Rhythm Society (HRS)/Asia Pacific Heart Rhythm Society (APHRS)/Latin American Heart Rhythm Society (LAHRS) expert consensus on risk assessment in cardiac arrhythmias: Use the right tool for the right outcome, in the right population. Europace. 2020; 22(8): 1147–8. DOI: 10.1093/europace/euaa065 32538434 PMC7400488

[11] Lloyd-Jones DM, Wang TJ, Leip EP, Larson MG, Levy D, Vasan RS, et al. Lifetime Risk for Development of Atrial Fibrillation. Circulation. 2004; 110(9): 1042–6. DOI: 10.1161/01.CIR.0000140263.20897.42 15313941

[12] Mou L, Norby FL, Chen LY, O’Neal WT, Lewis TT, Loehr LR, et al. Lifetime Risk of Atrial Fibrillation by Race and Socioeconomic Status: ARIC Study (Atherosclerosis Risk in Communities). Circ Arrhythm Electrophysiol. 2018; 11(7): e006350. DOI: 10.1161/CIRCEP.118.006350 30002066 PMC6053683

[13] Roth GA, Mensah GA, Johnson CO, Addolorato G, Ammirati E, Baddour LM, et al. Global Burden of Cardiovascular Diseases and Risk Factors, 1990–2019: Update From the GBD 2019 Study. J Am Coll Cardiol. 2020; 76(25): 2982–3021. DOI: 10.1016/j.jacc.2020.11.010 33309175 PMC7755038

[14] Zoni-Berisso M, Lercari F, Carazza T, Domenicucci S. Epidemiology of atrial fibrillation: European perspective. Clin Epidemiol. 2014; 6: 213–20. DOI: 10.2147/CLEP.S47385 24966695 PMC4064952

[15] Krijthe BP, Kunst A, Benjamin EJ, Lip GYH, Franco OH, Hofman A, et al. Projections on the number of individuals with atrial fibrillation in the European Union, from 2000 to 2060. Eur Heart J. 2013; 34(35): 2746–51. DOI: 10.1093/eurheartj/eht280 23900699 PMC3858024

[16] Colilla S, Crow A, Petkun W, Singer DE, Simon T, Liu X. Estimates of Current and Future Incidence and Prevalence of Atrial Fibrillation in the U.S. Adult Population. American Journal of Cardiology. 2013; 112(8): 1142–7. DOI: 10.1016/j.amjcard.2013.05.063 23831166

[17] Naser N, Dilic M, Durak A, Kulic M, Pepic E, Smajic E, et al. The Impact of Risk Factors and Comorbidities on The Incidence of Atrial Fibrillation. Mater Sociomed. 2017; 29(4): 231–6. DOI: 10.5455/msm.2017.29.231-236 29284990 PMC5723190

[18] Lowres N, Neubeck L, Redfern J, Freedman SB. Screening to identify unknown atrial fibrillation. A systematic review. Thromb Haemost. 2013; 110(2): 213–22. DOI: 10.1160/TH13-02-0165 23595785

[19] Lowres N, Olivier J, Chao TF, Chen SA, Chen Y, Diederichsen A, et al. Estimated stroke risk, yield, and number needed to screen for atrial fibrillation detected through single time screening: A multicountry patient-level meta-analysis of 141,220 screened individuals. PLoS Med. 2019; 16(9): e1002903. DOI: 10.1371/journal.pmed.1002903 31553733 PMC6760766

[20] Marcus GM, Alonso A, Peralta CA, Lettre G, Vittinghoff E, Lubitz SA, et al. European Ancestry as a Risk Factor for Atrial Fibrillation in African Americans. Circulation. 2010; 122(20): 2009–15. DOI: 10.1161/CIRCULATIONAHA.110.958306 21098467 PMC3058884

[21] Alonso A, Agarwal SK, Soliman EZ, Ambrose M, Chamberlain AM, Prineas RJ, et al. Incidence of atrial fibrillation in whites and African-Americans: The Atherosclerosis Risk in Communities (ARIC) study. Am Heart J. 2009; 158(1): 111–7. DOI: 10.1016/j.ahj.2009.05.010 19540400 PMC2720573

[22] Jensen PN, Thacker EL, Dublin S, Psaty BM, Heckbert SR. Racial Differences in the Incidence of and Risk Factors for Atrial Fibrillation in Older Adults: The Cardiovascular Health Study. J Am Geriatr Soc. 2013; 61(2): 276–80. DOI: 10.1111/jgs.12085 23320758 PMC3878638

[23] Dewland TA, Olgin JE, Vittinghoff E, Marcus GM. Incident Atrial Fibrillation Among Asians, Hispanics, Blacks, and Whites. Circulation. 2013; 128(23): 2470–7. DOI: 10.1161/CIRCULATIONAHA.113.002449 24103419

[24] Soni A, Earon A, Handorf A, Fahey N, Talati K, Bostrom J, et al. High Burden of Unrecognized Atrial Fibrillation in Rural India: An Innovative Community-Based Cross-Sectional Screening Program. JMIR public health and surveillance. 2016; 2(2): e159. DOI: 10.2196/publichealth.6517 27737818 PMC5083844

[25] Soni A, Karna S, Fahey N, Sanghai S, Patel H, Raithatha S, et al. Age-and-sex stratified prevalence of atrial fibrillation in rural Western India: Results of SMART-India, a population-based screening study. Int J Cardiol. 2019; 280: 84–8. DOI: 10.1016/j.ijcard.2018.12.016 30551905 PMC6378127

[26] Hingorani P, Natekar M, Deshmukh S, Karnad DR, Kothari S, Narula D, et al. Morphological abnormalities in baseline ECGs in healthy normal volunteers participating in phase I studies. Indian J Med Res. 2012; 135(3): 322–30. 22561618 PMC3361868

[27] Saggu DK, Sundar G, Nair SG, Bhargava VC, Lalukota K, Chennapragada S, et al. Prevalence of atrial fibrillation in an urban population in India: The Nagpur pilot study. Heart Asia. 2016; 8(1): 56–9. 10.1136/heartasia-2015-010674 PMC4898624 27326234

[28] Chan N-y, Choy C-c. Screening for atrial fibrillation in 13,122 Hong Kong citizens with smartphone electrocardiogram. Heart. 2017; 103(1): 24–31. DOI: 10.1136/heartjnl-2016-309993 27733533

[29] Noubiap JJ, Nyaga UF. A review of the epidemiology of atrial fibrillation in sub-Saharan Africa. J Cardiovasc Electrophysiol. 2019; 30(12): 3006–16. DOI: 10.1111/jce.14222 31596016

[30] Mandi DG, Bamouni J, Naïbé DT, Yaméogo RA, Kaboré E, Kambiré Y, et al. Epidemiology and long-term prognosis of atrial fibrillation in rural African patients. The Egyptian Heart Journal. 2019; 71(1): 6. DOI: 10.1186/s43044-019-0005-3 31659514 PMC6821409

[31] Gouda HN, Charlson F, Sorsdahl K, Ahmadzada S, Ferrari AJ, Erskine H, et al. Burden of non-communicable diseases in sub-Saharan Africa, 1990–2013; 2017: Results from the Global Burden of Disease Study 2017. The Lancet Global Health. 2019; 7(10): e1375–e87. DOI: 10.1016/S2214-109X(19)30374-2 31537368

[32] Lau C-P, Gbadebo TD, Connolly SJ, Van Gelder IC, Capucci A, Gold MR, et al. Ethnic Differences in Atrial Fibrillation Identified Using Implanted Cardiac Devices. J Cardiovasc Electrophysiol. 2013; 24(4): 381–7. DOI: 10.1111/jce.12066 23356818

[33] Muthalaly RG, Koplan BA, Albano A, North C, Campbell JI, Kakuhikire B, et al. Low population prevalence of atrial fibrillation in rural Uganda: A community-based cross-sectional study. Int J Cardiol. 2018; 271: 87–91. DOI: 10.1016/j.ijcard.2018.05.074 29859712 PMC6143408

[34] Paixão GMM, Silva LGS, Gomes PR, Lima EM, Ferreira MPF, Oliveira DM, et al. Evaluation of Mortality in Atrial Fibrillation: Clinical Outcomes in Digital Electrocardiography (CODE) Study. Glob Heart. 2020; 15(1): 48. DOI: 10.5334/gh.772 32923342 PMC7413140

[35] Marcolino MS, Palhares DM, Benjamin EJ, Ribeiro AL. Atrial fibrillation: prevalence in a large database of primary care patients in Brazil. Europace. 2015; 17(12): 1787–90. DOI: 10.1093/europace/euv185 26056188 PMC4700731

[36] Mora-Llabata DD-MV, Roldán-Torres I, Mateu-Navarro C, Sanz-García JJ, Moreno-Ballester V, Mira-Gimeno S, Albiñana-Fernández F. Prevalencia de fibrilación auricular y características de la fibrilación auricular no valvular en la población general. Revista Colombiana de Cardiología. 2017; 24(1): 26–33. DOI: 10.1016/j.rccar.2016.03.021

[37] Cubillos L, Haddad A, Kuznik A, Mould-Quevedo J. Burden of disease from atrial fibrillation in adults from seven countries in Latin America. Int J Gen Med. 2014; 7: 441–8. DOI: 10.2147/IJGM.S62819 25214802 PMC4159313

[38] Joseph PG, Healey JS, Raina P, Connolly SJ, Ibrahim Q, Gupta R, et al. Global variations in the prevalence, treatment, and impact of atrial fibrillation in a multi-national cohort of 153,152 middle-aged individuals. Cardiovasc Res; 2020. DOI: 10.1093/cvr/cvaa241 32777820

[39] Kakkar AK, Mueller I, Bassand JP, Fitzmaurice DA, Goldhaber SZ, Goto S, et al. Risk profiles and antithrombotic treatment of patients newly diagnosed with atrial fibrillation at risk of stroke: Perspectives from the international, observational, prospective GARFIELD registry. PLoS One. 2013; 8(5): e63479. DOI: 10.1371/journal.pone.0063479 23704912 PMC3660389

[40] Goto S, Oh S, Cools F, Koretsune Y, Angchaisuksiri P, Rushton-Smith S, et al. Regional differences in use of antithrombotic therapy for stroke prevention in atrial fibrillation and associated outcomes: *European and Asian insights*. Eur Heart J. 2013; 34: P4277. DOI: 10.1093/eurheartj/eht309.P4277

[41] Nguyen TN, Hilmer SN, Cumming RG. Review of epidemiology and management of atrial fibrillation in developing countries. Int J Cardiol. 2013; 167(6): 2412–20. DOI: 10.1016/j.ijcard.2013.01.184 23453870

[42] Narasimhan C, Verma JS, Ravi Kishore AG, Singh B, Dani S, Chawala K, et al. Cardiovascular risk profile and management of atrial fibrillation in India: Real world data from RealiseAF survey. Indian Heart J. 2016; 68(5): 663–70. DOI: 10.1016/j.ihj.2015.12.011 27773405 PMC5079132

[43] Oldgren J, Healey JS, Ezekowitz M, Commerford P, Avezum A, Pais P, et al. Variations in cause and management of atrial fibrillation in a prospective registry of 15,400 emergency department patients in 46 countries: The RE-LY Atrial Fibrillation Registry. Circulation. 2014; 129(15): 1568–76. DOI: 10.1161/CIRCULATIONAHA.113.005451 24463370

[44] Benjamin EJ, Wolf PA, D’Agostino RB, Silbershatz H, Kannel WB, Levy D. Impact of Atrial Fibrillation on the Risk of Death. Circulation. 1998; 98(10): 946–52. DOI: 10.1161/01.CIR.98.10.946 9737513

[45] Stewart S, Hart CL, Hole DJ, McMurray JJV. A population-based study of the long-term risks associated with atrial fibrillation: 20-year follow-up of the Renfrew/Paisley study. Am J Med. 2002; 113(5): 359–64. DOI: 10.1016/S0002-9343(02)01236-6 12401529

[46] Andersson T, Magnuson A, Bryngelsson I-L, Frøbert O, Henriksson KM, Edvardsson N, et al. All-cause mortality in 272 186 patients hospitalized with incident atrial fibrillation 1995–2008: A Swedish nationwide long-term case-control study. Eur Heart J. 2013; 34(14): 1061–7. DOI: 10.1093/eurheartj/ehs469 23321349 PMC3618889

[47] Sepehri Shamloo A, Dagres N, Müssigbrodt A, Stauber A, Kircher S, Richter S, et al. Atrial Fibrillation and Cognitive Impairment: New Insights and Future Directions. Heart Lung Circ. 2020; 29(1): 69–85. DOI: 10.1016/j.hlc.2019.05.185 31262618

[48] Benjamin EJ, Virani SS, Callaway CW, Chamberlain AM, Chang AR, Cheng S, et al. Heart Disease and Stroke Statistics 2014; 2018 Update: A Report From the American Heart Association. Circulation. 2018; 137(12): e67–e492. DOI: 10.1161/CIR.0000000000000573 29386200

[49] PricewaterhouseCoopers. Commissioned by the National Stroke Foundation. The Economic Costs of Atrial Fibrillation in Australia. 2010.25/6/2020. Available from: http://www.hps.com.au/wp-content/uploads/2016/10/Economic-costs-of-atrial-fibrillation-in-Australia.pdf.

[50] Freedman B, Camm J, Calkins H, Healey JS, Rosenqvist M, Wang J, et al. Screening for Atrial Fibrillation: A Report of the AF-SCREEN International Collaboration. Circulation. 2017; 135(19): 1851–67. DOI: 10.1161/CIRCULATIONAHA.116.026693 28483832

[51] Schnabel RB, Yin X, Gona P, Larson MG, Beiser AS, McManus DD, et al. 50-year trends in atrial fibrillation prevalence, incidence, risk factors, and mortality in the Framingham Heart Study: A cohort study. The Lancet. 2015; 386(9989): 154–62. DOI: 10.1016/S0140-6736(14)61774-8 PMC4553037 25960110

[52] Menezes AR, Lavie CJ, De Schutter A, Milani RV, O’Keefe J, DiNicolantonio JJ, et al. Lifestyle modification in the prevention and treatment of atrial fibrillation. J Progress in cardiovascular diseases. 2015; 58(2): 117–25. DOI: 10.1016/j.pcad.2015.07.001 26184674

[53] Du X, Dong J, Ma C. Is atrial fibrillation a preventable disease? J Am Coll Cardiol. 2017; 69(15): 1968–82. DOI: 10.1016/j.jacc.2017.02.020 28408027

[54] Ahmadi SS, Svensson A-M, Pivodic A, Rosengren A, Lind M. Risk of atrial fibrillation in persons with type 2 diabetes and the excess risk in relation to glycaemic control and renal function: A Swedish cohort study. J Cardiovascular diabetology. 2020; 19(1): 1–12. DOI: 10.1186/s12933-019-0983-1 PMC6969407 31954408

[55] Murphy NF, Simpson CR, Jhund PS, Stewart S, Kirkpatrick M, Chalmers J, et al. A national survey of the prevalence, incidence, primary care burden and treatment of atrial fibrillation in Scotland. J Heart. 2007; 93(5): 606–12. DOI: 10.1136/hrt.2006.107573 PMC1955558 17277353

[56] Misialek JR, Rose KM, Everson-Rose SA, Soliman EZ, Clark CJ, Lopez FL, et al. Socioeconomic status and the incidence of atrial fibrillation in whites and blacks: The Atherosclerosis Risk in Communities (ARIC) study. J Am Heart Assoc. 2014; 3(4): e001159. DOI: 10.1161/JAHA.114.001159 25142059 PMC4310413

[57] Wändell P, Carlsson AC, Gasevic D, Holzmann MJ, Ärnlöv J, Sundquist J, et al. Socioeconomic factors and mortality in patients with atrial fibrillation—a cohort study in Swedish primary care. European J of public health. 2018; 28(6): 1103–9. DOI: 10.1093/eurpub/cky075 29746622 PMC6241208

[58] Pathak RK, Middeldorp ME, Lau DH, Mehta AB, Mahajan R, Twomey D, et al. Aggressive risk factor reduction study for atrial fibrillation and implications for the outcome of ablation: The ARREST-AF cohort study. J Am Coll Cardiol. 2014; 64(21): 2222–31. DOI: 10.1016/j.jacc.2014.09.028 25456757

[59] Abed HS, Wittert GA, Leong DP, Shirazi MG, Bahrami B, Middeldorp ME, et al. Effect of weight reduction and cardiometabolic risk factor management on symptom burden and severity in patients with atrial fibrillation: A randomized clinical trial. JAMA. 2013; 310(19): 2050–60. DOI: 10.1001/jama.2013.280521 24240932

[60] Pathak RK, Middeldorp ME, Meredith M, Mehta AB, Mahajan R, Wong CX, et al. Long-Term Effect of Goal-Directed Weight Management in an Atrial Fibrillation Cohort: A Long-Term Follow-Up Study (LEGACY). J Am Coll Cardiol. 2015; 65(20): 2159–69. DOI: 10.1016/j.jacc.2015.03.002 25792361

[61] Karjalainen J, Kujala UM, Kaprio J, Sarna S, Viitasalo M. Lone atrial fibrillation in vigorously exercising middle aged men: case-control study. BMJ. 1998; 316(7147): 1784–5. DOI: 10.1136/bmj.316.7147.1784 9624065 PMC28577

[62] Baldesberger S, Bauersfeld U, Candinas R, Seifert B, Zuber M, Ritter M, et al. Sinus node disease and arrhythmias in the long-term follow-up of former professional cyclists. Eur Heart J. 2008; 29(1): 71–8. DOI: 10.1093/eurheartj/ehm555 18065754

[63] Molina L, Mont L, Marrugat J, Berruezo A, Brugada J, Bruguera J, et al. Long-term endurance sport practice increases the incidence of lone atrial fibrillation in men: a follow-up study. Europace. 2008; 10(5): 618–23. DOI: 10.1093/europace/eun071 18390875

[64] Nielsen JR, Wachtell K, Abdulla J. The Relationship Between Physical Activity and Risk of Atrial Fibrillation-A Systematic Review and Meta-Analysis. J Atr Fibrillation. 2013; 5(5): 789. 28496815 10.4022/jafib.789 PMC5153110

[65] Epstein LJ, Kristo D, Strollo PJ, Jr., Friedman N, Malhotra A, Patil SP, et al. Clinical guideline for the evaluation, management and long-term care of obstructive sleep apnea in adults. J Clin Sleep Med. 2009; 5(3): 263–76. DOI: 10.5664/jcsm.27497 19960649 PMC2699173

[66] Linz D, McEvoy RD, Cowie MR, Somers VK, Nattel S, Levy P, et al. Associations of Obstructive Sleep Apnea With Atrial Fibrillation and Continuous Positive Airway Pressure Treatment: A Review. JAMA Cardiol. 2018; 3(6): 532–40. DOI: 10.1001/jamacardio.2018.0095 29541763

[67] Kotseva K, De Backer G, De Bacquer D, Rydén L, Hoes A, Grobbee D, et al. Lifestyle and impact on cardiovascular risk factor control in coronary patients across 27 countries: Results from the European Society of Cardiology ESC-EORP EUROASPIRE V registry. Eur J Prev Cardiol. 2019; 26(8): 824–35. DOI: 10.1177/2047487318825350 30739508

[68] Proia KK, Thota AB, Njie GJ, Finnie RK, Hopkins DP, Mukhtar Q, et al. Team-based care and improved blood pressure control: a community guide systematic review. Am J Prev Med. 2014; 47(1): 86–99. DOI: 10.1016/j.amepre.2014.03.004 24933494 PMC4672378

[69] Buhse S, Mühlhauser I, Heller T, Kuniss N, Müller UA, Kasper J, et al. Informed shared decision-making programme on the prevention of myocardial infarction in type 2 diabetes: a randomised controlled trial. BMJ open. 2015; 5(11): e009116. DOI: 10.1136/bmjopen-2015-009116 PMC4654390 26567256

[70] Arnett DK, Blumenthal RS, Albert MA, Buroker AB, Goldberger ZD, Hahn EJ, et al. 2019 ACC/AHA Guideline on the Primary Prevention of Cardiovascular Disease: A Report of the American College of Cardiology/American Heart Association Task Force on Clinical Practice Guidelines. J Am Coll Cardiol. 2019; 74(10): e177–e232. 30894318 10.1016/j.jacc.2019.03.010 PMC7685565

[71] Johansson C, Dahlqvist E, Andersson J, Jansson JH, Johansson L. Incidence, type of atrial fibrillation and risk factors for stroke: A population-based cohort study. Clin Epidemiol. 2017; 9: 53–62. DOI: 10.2147/CLEP.S122916 28182159 PMC5283072

[72] Healey JS, Alings M, Ha A, Leong-Sit P, Birnie DH, de Graaf JJ, et al. Subclinical Atrial Fibrillation in Older Patients. Circulation. 2017; 136(14): 1276–83. DOI: 10.1161/CIRCULATIONAHA.117.028845 28778946

[73] Hindricks G, Potpara T, Dagres N, Arbelo E, Bax JJ, Blomström-Lundqvist C, et al. 2020 ESC Guidelines for the diagnosis and management of atrial fibrillation developed in collaboration with the European Association of Cardio-Thoracic Surgery (EACTS). Eur Heart J; 2020. 10.1093/eurheartj/ehab648 34520521

[74] Ganesan AN, Chew DP, Hartshorne T, Selvanayagam JB, Aylward PE, Sanders P, et al. The impact of atrial fibrillation type on the risk of thromboembolism, mortality, and bleeding: A systematic review and meta-analysis. Eur Heart J. 2016; 37(20): 1591–602. DOI: 10.1093/eurheartj/ehw007 26888184

[75] Israel CW, Grönefeld G, Ehrlich JR, Li YG, Hohnloser SH. Long-term risk of recurrent atrial fibrillation as documented by an implantable monitoring device: Implications for optimal patient care. J Am Coll Cardiol. 2004; 43(1): 47–52. DOI: 10.1016/j.jacc.2003.08.027 14715182

[76] Flaker GC, Belew K, Beckman K, Vidaillet H, Kron J, Safford R, et al. Asymptomatic atrial fibrillation: Demographic features and prognostic information from the Atrial Fibrillation Follow-up Investigation of Rhythm Management (AFFIRM) study. Am Heart J. 2005; 149(4): 657–63. DOI: 10.1016/j.ahj.2004.06.032 15990749

[77] Boriani G, Laroche C, Diemberger I, Fantecchi E, Popescu MI, Rasmussen LH, et al. Asymptomatic atrial fibrillation: clinical correlates, management, and outcomes in the EORP-AF Pilot General Registry. Am J Med. 2015; 128(5): 509–18.e2. DOI: 10.1016/j.amjmed.2014.11.026 25534423

[78] Banerjee A, Taillandier S, Olesen JB, Lane DA, Lallemand B, Lip GY, et al. Pattern of atrial fibrillation and risk of outcomes: the Loire Valley Atrial Fibrillation Project. Int J Cardiol. 2013; 167(6): 2682–7. DOI: 10.1016/j.ijcard.2012.06.118 22795403

[79] Jabaudon D, Sztajzel J, Sievert K, Landis T, Sztajzel R. Usefulness of ambulatory 7-day ECG monitoring for the detection of atrial fibrillation and flutter after acute stroke and transient ischemic attack. Stroke. 2004; 35(7): 1647–51. DOI: 10.1161/01.STR.0000131269.69502.d9 15155965

[80] Edwards SJ, Wakefield V, Jhita T, Kew K, Cain P, Marceniuk G. Implantable cardiac monitors to detect atrial fibrillation after cryptogenic stroke: a systematic review and economic evaluation. Health Technol Assess. 2020; 24(5): 1–184. DOI: 10.3310/hta24050 PMC6983910 31944175

[81] Schnabel RB, Haeusler KG, Healey JS, Freedman B, Boriani G, Brachmann J, et al. Searching for Atrial Fibrillation Poststroke: A White Paper of the AF-SCREEN International Collaboration. Circulation. 2019; 140(22): 1834–50. DOI: 10.1161/CIRCULATIONAHA.119.040267 31765261

[82] Perez MV, Mahaffey KW, Hedlin H, Rumsfeld JS, Garcia A, Ferris T, et al. Large-Scale Assessment of a Smartwatch to Identify Atrial Fibrillation. N Engl J Med. 2019; 381(20): 1909–17. DOI: 10.1056/NEJMoa1901183 31722151 PMC8112605

[83] Brasier N, Raichle CJ, Dörr M, Becke A, Nohturfft V, Weber S, et al. Detection of atrial fibrillation with a smartphone camera: first prospective, international, two-centre, clinical validation study (DETECT AF PRO). Europace. 2019; 21(1): 41–7. DOI: 10.1093/europace/euy176 30085018 PMC6321964

[84] Guo Y, Wang H, Zhang H, Liu T, Liang Z, Xia Y, et al. Mobile Photoplethysmographic Technology to Detect Atrial Fibrillation. J Am Coll Cardiol. 2019; 74(19): 2365–75. DOI: 10.1016/j.jacc.2019.08.019 31487545

[85] Castaneda D, Esparza A, Ghamari M, Soltanpur C, Nazeran H. A review on wearable photoplethysmography sensors and their potential future applications in health care. Int J Biosens Bioelectron. 2018; 4(4): 195–202. DOI: 10.15406/ijbsbe.2018.04.00125 30906922 PMC6426305

[86] Dörr M, Nohturfft V, Brasier N, Bosshard E, Djurdjevic A, Gross S, et al. The WATCH AF Trial: SmartWATCHes for Detection of Atrial Fibrillation. JACC Clin Electrophysiol. 2019; 5(2): 199–208. DOI: 10.1016/j.jacep.2018.10.006 30784691

[87] Perez MV, Mahaffey KW, Hedlin H, Rumsfeld JS, Garcia A, Ferris T, et al. Large-Scale Assessment of a Smartwatch to Identify Atrial Fibrillation. N Engl J Med. 2019; 381(20): 1909–17. DOI: 10.1056/NEJMoa1901183 31722151 PMC8112605

[88] Cheung CC, Gin KG, Andrade JG. Watch Out: The Many Limitations in Smartwatch-Driven AF Detection. JACC Clin Electrophysiol. 2019; 5(4): 525–6. DOI: 10.1016/j.jacep.2019.02.008 31000110

[89] Statista. Wearable user penetration rate in the United States, in 2017, by age; 2020.

[90] National Institute for Health and Care Excellence (NICE). WatchBP Home A for opportunistically detecting atrial fibrillation during diagnosis and monitoring of hypertension; 2013: https://www.nice.org.uk/guidance/mtg13.

[91] Yan BP, Lai WHS, Chan CKY, Au ACK, Freedman B, Poh YC, et al. High-Throughput, Contact-Free Detection of Atrial Fibrillation From Video With Deep Learning. JAMA Cardiol. 2020; 5(1): 105–7. DOI: 10.1001/jamacardio.2019.4004 31774461 PMC6902123

[92] Nijasri C, Suwanwela AC, Autjimanon H, Ounahachok T, Decha-umphai C, Chockchai S, et al. Atrial fibrillation prevalence and risk profile from novel community-based screening in Thailand: A prospective multi-centre study. IJC Heart & Vasculature. 2021; 32. DOI: 10.1016/j.ijcha.2020.100709 PMC7811109 33490362

[93] Behzadi A, Sepehri Shamloo A, Mouratis K, Hindricks G, Arya A, Bollmann A. Feasibility and Reliability of SmartWatch to Obtain 3-Lead Electrocardiogram Recordings. Sensors (Basel). 2020; 20(18). DOI: 10.3390/s20185074 PMC7571061 32906661

[94] Bumgarner JM, Lambert CT, Hussein AA, Cantillon DJ, Baranowski B, Wolski K, et al. Smartwatch Algorithm for Automated Detection of Atrial Fibrillation. J Am Coll Cardiol. 2018; 71(21): 2381–8. DOI: 10.1016/j.jacc.2018.03.003 29535065

[95] Gwynn J, Gwynne K, Rodrigues R, Thompson S, Bolton G, Dimitropoulos Y, et al. Atrial Fibrillation in Indigenous Australians: A Multisite Screening Study Using a Single-Lead ECG Device in Aboriginal Primary Health Settings. Heart Lung Circ; 2020. DOI: 10.1016/j.hlc.2020.06.009 32807629

[96] Svennberg E, Engdahl J, Al-Khalili F, Friberg L, Frykman V, Rosenqvist M. Mass Screening for Untreated Atrial Fibrillation: The Strokestop Study. Circulation. 2015; 131(25): 2176–84. DOI: 10.1161/CIRCULATIONAHA.114.014343 25910800

[97] Diamantino AC, Nascimento BR, Beaton AZ, Nunes MCP, Oliveira KKB, Rabelo LC, et al. Atrial fibrillation detection with a portable device during cardiovascular screening in primary care. Heart. 2020; 106(16): 1261–6. DOI: 10.1136/heartjnl-2019-316277 32019822

[98] Silver LSA, Johnson C, Taylor K, Jiang J, Anderson M, Rainie L. Mobile Connectivity in Emerging Economies Pew Research Center; 2019.

[99] Communications TGSfM. The Mobile Economy 2020; 2020.

[100] Boriani G, Schnabel RB, Healey JS, Lopes RD, Verbiest-van Gurp N, Lobban T, et al. Consumer-led screening for atrial fibrillation using consumer-facing wearables, devices, and apps: A survey of health care professionals by AF-SCREEN international collaboration. Eur J Intern Med; 2020. DOI: 10.1016/j.ejim.2020.09.005 32933842

[101] Lopez-Jimenez F, Attia Z, Arruda-Olson AM, Carter R, Chareonthaitawee P, Jouni H, et al. Artificial Intelligence in Cardiology: Present and Future. Mayo Clin Proc. 2020; 95(5): 1015–39. DOI: 10.1016/j.mayocp.2020.01.038 32370835

[102] Attia ZI, Noseworthy PA, Lopez-Jimenez F, Asirvatham SJ, Deshmukh AJ, Gersh BJ, et al. An artificial intelligence-enabled ECG algorithm for the identification of patients with atrial fibrillation during sinus rhythm: A retrospective analysis of outcome prediction. Lancet. 2019; 394(10201): 861–7. DOI: 10.1016/S0140-6736(19)31721-0 31378392

[103] Kashou AH, Rabinstein AA, Attia IZ, Asirvatham SJ, Gersh BJ, Friedman PA, et al. Recurrent cryptogenic stroke: A potential role for an artificial intelligence-enabled electrocardiogram? HeartRhythm Case Rep. 2020; 6(4): 202–5. DOI: 10.1016/j.hrcr.2019.12.013 32322497 PMC7156980

[104] Hart RG, Sharma M, Mundl H, Kasner SE, Bangdiwala SI, Berkowitz SD, et al. Rivaroxaban for Stroke Prevention after Embolic Stroke of Undetermined Source. N Engl J Med. 2018; 378(23): 2191–201. DOI: 10.1056/NEJMoa1802686 29766772

[105] Diener HC, Sacco RL, Easton JD, Granger CB, Bernstein RA, Uchiyama S, et al. Dabigatran for Prevention of Stroke after Embolic Stroke of Undetermined Source. N Engl J Med. 2019; 380(20): 1906–17. DOI: 10.1056/NEJMoa1813959 31091372

[106] Noseworthy PA, Attia ZI, Brewer LC, Hayes SN, Yao X, Kapa S, et al. Assessing and Mitigating Bias in Medical Artificial Intelligence: The Effects of Race and Ethnicity on a Deep Learning Model for ECG Analysis. Circ Arrhythm Electrophysiol. 2020; 13(3): e007988. DOI: 10.1161/CIRCEP.119.007988 32064914 PMC7158877

[107] Steinberg JS, Varma N, Cygankiewicz I, Aziz P, Balsam P, Baranchuk A, et al. 2017 ISHNE-HRS expert consensus statement on ambulatory ECG and external cardiac monitoring/telemetry. Heart Rhythm. 2017; 14(7): e55–e96. DOI: 10.1016/j.hrthm.2017.03.038 28495301

[108] Steinhubl SR, Waalen J, Edwards AM, Ariniello LM, Mehta RR, Ebner GS, et al. Effect of a Home-Based Wearable Continuous ECG Monitoring Patch on Detection of Undiagnosed Atrial Fibrillation: The mSToPS Randomized Clinical Trial. JAMA. 2018; 320(2): 146–55. DOI: 10.1001/jama.2018.8102 29998336 PMC6583518

[109] Gladstone DJ, Spring M, Dorian P, Panzov V, Thorpe KE, Hall J, et al. Atrial fibrillation in patients with cryptogenic stroke. N Engl J Med. 2014; 370(26): 2467–77. DOI: 10.1056/NEJMoa1311376 24963566

[110] Sanna T, Diener HC, Passman RS, Di Lazzaro V, Bernstein RA, Morillo CA, et al. Cryptogenic stroke and underlying atrial fibrillation. N Engl J Med. 2014; 370(26): 2478–86. DOI: 10.1056/NEJMoa1313600 24963567

[111] Diederichsen SZ, Haugan KJ, Kronborg C, Graff C, Højberg S, Køber L, et al. Comprehensive Evaluation of Rhythm Monitoring Strategies in Screening for Atrial Fibrillation: Insights From Patients at Risk Monitored Long Term With an Implantable Loop Recorder. Circulation. 2020; 141(19): 1510–22. DOI: 10.1161/CIRCULATIONAHA.119.044407 32114796

[112] Chen Y, Huang QF, Sheng CS, Zhang W, Shao S, Wang D, et al. Detection rate and treatment gap for atrial fibrillation identified through screening in community health centers in China (AF-CATCH): A prospective multicenter study. PLoS Med. 2020; 17(7): e1003146. DOI: 10.1371/journal.pmed.1003146 32673305 PMC7365395

[113] Orchard J, Neubeck L, Freedman B, Li J, Webster R, Zwar N, et al. eHealth Tools to Provide Structured Assistance for Atrial Fibrillation Screening, Management, and Guideline-Recommended Therapy in Metropolitan General Practice: The AF – SMART Study. J Am Heart Assoc. 2019; 8(1): e010959. DOI: 10.1161/JAHA.118.010959 30590964 PMC6405712

[114] Tarride J-E, Dolovich L, Blackhouse G, Guertin JR, Burke N, Manja V, et al. Screening for atrial fibrillation in Canadian pharmacies: an economic evaluation. CMAJ open. 2017; 5(3): E653–E61. DOI: 10.9778/cmajo.20170042 PMC5621947 28835370

[115] Alkmim MB, Figueira RM, Marcolino MS, Cardoso CS, Pena de Abreu M, Cunha LR, et al. Improving patient access to specialized health care: the Telehealth Network of Minas Gerais, Brazil. Bull World Health Organ. 2012; 90(5): 373–8. DOI: 10.2471/BLT.11.099408 22589571 PMC3341691

[116] Bunch TJ, Steinberg BA. Revisiting Rate versus Rhythm Control in Atrial Fibrillation — Timing Matters. N Engl J Med; 2020. DOI: 10.1056/NEJMe2027180 32865379

[117] Kirchhof P, Camm AJ, Goette A, Brandes A, Eckardt L, Elvan A, et al. Early Rhythm-Control Therapy in Patients with Atrial Fibrillation. N Engl J Med; 2020. DOI: 10.1056/NEJMoa2019422

[118] Lip GY, Nieuwlaat R, Pisters R, Lane DA, Crijns HJ. Refining clinical risk stratification for predicting stroke and thromboembolism in atrial fibrillation using a novel risk factor-based approach: The euro heart survey on atrial fibrillation. Chest. 2010; 137(2): 263–72. DOI: 10.1378/chest.09-1584 19762550

[119] Borre ED, Goode A, Raitz G, Shah B, Lowenstern A, Chatterjee R, et al. Predicting Thromboembolic and Bleeding Event Risk in Patients with Non-Valvular Atrial Fibrillation: A Systematic Review. Thromb Haemost. 2018; 118(12): 2171–87. DOI: 10.1055/s-0038-1675400 30376678 PMC6754740

[120] Al-Khatib SM, Allen LaPointe NM, Chatterjee R, Crowley MJ, Dupre ME, Kong DF, et al. Rate- and rhythm-control therapies in patients with atrial fibrillation: A systematic review. Ann Intern Med. 2014; 160(11): 760–73. DOI: 10.7326/M13-1467 24887617

[121] Tamariz LJ, Bass EB. Pharmacological rate control of atrial fibrillation. Cardiol Clin. 2004; 22(1): 35–45. DOI: 10.1016/S0733-8651(03)00111-5 14994846

[122] Nikolaidou T, Channer KS. Chronic atrial fibrillation: A systematic review of medical heart rate control management. Postgrad Med J. 2009; 85(1004): 303–12. DOI: 10.1136/pgmj.2008.068908 19528305

[123] Marshall DA, Levy AR, Vidaillet H, Fenwick E, Slee A, Blackhouse G, et al. Cost-effectiveness of rhythm versus rate control in atrial fibrillation. Ann Intern Med. 2004; 141(9): 653–61. DOI: 10.7326/0003-4819-141-9-200411020-00005 15520421

[124] Mkoko P, Bahiru E, Ajijola OA, Bonny A, Chin A. Cardiac arrhythmias in low- and middle-income countries. Cardiovasc Diagn Ther. 2020; 10(2): 350–60. DOI: 10.21037/cdt.2019.09.21 32420117 PMC7225444

[125] Calkins H, Hindricks G, Cappato R, Kim YH, Saad EB, Aguinaga L, et al. HRS/EHRA/ECAS/APHRS/SOLAECE expert consensus statement on catheter and surgical ablation of atrial fibrillation. Heart Rhythm. 2017; 14(10): e275–e444. 28506916 10.1016/j.hrthm.2017.05.012 PMC6019327

[126] Middeldorp ME, Ariyaratnam J, Lau D, Sanders P. Lifestyle modifications for treatment of atrial fibrillation. Heart. 2020; 106(5): 325–32. DOI: 10.1136/heartjnl-2019-315327 31712316

[127] Hindricks G, Potpara T, Dagres N, Arbelo E, Bax JJ, Blomstrom-Lundqvist C, et al. 2020 ESC Guidelines for the diagnosis and management of atrial fibrillation developed in collaboration with the European Association of Cardio-Thoracic Surgery (EACTS). Eur Heart J; 2020. 10.1093/eurheartj/ehab648 34520521

[128] Camm AJ, Capucci A, Hohnloser SH, Torp-Pedersen C, Van Gelder IC, Mangal B, et al. A randomized active-controlled study comparing the efficacy and safety of vernakalant to amiodarone in recent-onset atrial fibrillation. J Am Coll Cardiol. 2011; 57(3): 313–21. DOI: 10.1016/j.jacc.2010.07.046 21232669

[129] Markey GC, Salter N, Ryan J. Intravenous Flecainide for Emergency Department Management of Acute Atrial Fibrillation. J Emerg Med. 2018; 54(3): 320–7. DOI: 10.1016/j.jemermed.2017.11.016 29269083

[130] Reisinger J, Gatterer E, Lang W, Vanicek T, Eisserer G, Bachleitner T, et al. Flecainide versus ibutilide for immediate cardioversion of atrial fibrillation of recent onset. Eur Heart J. 2004; 25(15): 1318–24. DOI: 10.1016/j.ehj.2004.04.030 15288159

[131] Zhang N, Guo JH, Zhang H, Li XB, Zhang P, Xn Y. Comparison of intravenous ibutilide vs. propafenone for rapid termination of recent onset atrial fibrillation. Int J Clin Pract. 2005; 59(12): 1395–400. DOI: 10.1111/j.1368-5031.2005.00705.x 16351670

[132] Roy D, Pratt CM, Torp-Pedersen C, Wyse DG, Toft E, Juul-Moller S, et al. Vernakalant hydrochloride for rapid conversion of atrial fibrillation: a phase 3, randomized, placebo-controlled trial. Circulation. 2008; 117(12): 1518–25. DOI: 10.1161/CIRCULATIONAHA.107.723866 18332267

[133] Conde D, Costabel JP, Caro M, Ferro A, Lambardi F, Corrales Barboza A, et al. Flecainide versus vernakalant for conversion of recent-onset atrial fibrillation. Int J Cardiol. 2013; 168(3): 2423–5. DOI: 10.1016/j.ijcard.2013.02.006 23518212

[134] Martinez-Marcos FJ, Garcia-Garmendia JL, Ortega-Carpio A, Fernandez-Gomez JM, Santos JM, Camacho C. Comparison of intravenous flecainide, propafenone, and amiodarone for conversion of acute atrial fibrillation to sinus rhythm. Am J Cardiol. 2000; 86(9): 950–3. DOI: 10.1016/S0002-9149(00)01128-0 11053705

[135] Arbelo E, Brugada J, Hindricks G, Maggioni A, Tavazzi L, Vardas P, et al. ESC-EURObservational Research Programme: The Atrial Fibrillation Ablation Pilot Study, conducted by the European Heart Rhythm Association. Europace. 2012; 14(8): 1094–103. DOI: 10.1093/europace/eus153 22628450

[136] Arbelo E, Brugada J, Hindricks G, Maggioni AP, Tavazzi L, Vardas P, et al. The atrial fibrillation ablation pilot study: A European Survey on Methodology and results of catheter ablation for atrial fibrillation conducted by the European Heart Rhythm Association. Eur Heart J. 2014; 35(22): 1466–78. DOI: 10.1093/eurheartj/ehu001 24487524

[137] Arbelo E, Brugada J, Blomstrom-Lundqvist C, Laroche C, Kautzner J, Pokushalov E, et al. Contemporary management of patients undergoing atrial fibrillation ablation: in-hospital and 1-year follow-up findings from the ESC-EHRA atrial fibrillation ablation long-term registry. Eur Heart J. 2017; 38(17): 1303–16. DOI: 10.1093/eurheartj/ehw564 28104790

[138] Calkins H, Hindricks G, Cappato R, Kim YH, Saad EB, Aguinaga L, et al. 2017 HRS/EHRA/ECAS/APHRS/SOLAECE expert consensus statement on catheter and surgical ablation of atrial fibrillation: Executive summary. Europace. 2018; 20(1): 157–208. DOI: 10.1093/europace/eux275 29016841 PMC5892164

[139] Hindricks G, Sepehri Shamloo A, Lenarczyk R, Kalarus Z, Arya A, Kircher S, et al. Catheter ablation of atrial fibrillation: Current status, techniques, outcomes and challenges. Kardiol Pol. 2018; 76(12): 1680–6. DOI: 10.5603/KP.a2018.0216 30406938

[140] Keegan R, Aguinaga L, Fenelon G, Uribe W, Rodriguez Diez G, Scanavacca M, et al. The first Latin American Catheter Ablation Registry. Europace. 2015; 17(5): 794–800. DOI: 10.1093/europace/euu322 25616407

[141] Keegan R, et al. Catheter ablation of atrial fibrillation in Latin America: Results of the first registry of the Latin American Society of Pacing and Electrophysiology Cardiac (SOLAECE). RevUrugCardiol. 2016; 31(1): 165–75.

[142] Bonny A, Ngantcha M, Scholtz W, Chin A, Nel G, Anzouan-Kacou J-B, et al. Cardiac Arrhythmias in Africa: Epidemiology, Management Challenges, and Perspectives. Journal of the American College of Cardiology. 2019; 73(1): 100–9. DOI: 10.1016/j.jacc.2018.09.084 30621939

[143] Bonny A, Ngantcha M, Yuyun M, Karaye KM, Scholtz W, Suliman A, et al. Cardiac arrhythmia services in Africa from 2011 to 2018: The second report from the Pan African Society of Cardiology working group on cardiac arrhythmias and pacing. Europace. 2020; 22(3): 420–33. DOI: 10.1093/europace/euz354 31989158

[144] Bonny A, Ngantcha M, Yuyun MF, Karaye KM, Scholtz W, Suliman A, et al. Cardiac arrhythmia services in Africa from 2011 to 2018: The second report from the Pan African Society of Cardiology working group on cardiac arrhythmias and pacing. EP Europace. 2020; 22(3): 420–33. DOI: 10.1093/europace/euz354 31989158

[145] Ntep-Gweth M, Zimmermann M, Meiltz A, Kingue S, Ndobo P, Urban P, et al. Atrial fibrillation in Africa: clinical characteristics, prognosis, and adherence to guidelines in Cameroon. Europace: European pacing, arrhythmias, and cardiac electrophysiology: journal of the working groups on cardiac pacing, arrhythmias, and cardiac cellular electrophysiology of the European Society of Cardiology. 2010; 12(4): 482–7. DOI: 10.1093/europace/euq006 20179174

[146] Ettarh R. Patterns of international collaboration in cardiovascular research in sub-Saharan Africa. Cardiovascular Journal of Africa. 2016; 27(3): 194. DOI: 10.5830/CVJA-2015-082 27841904 PMC5101473

[147] Mkoko P, Bahiru E, Ajijola OA, Bonny A, Chin A. Cardiac arrhythmias in low-and middle-income countries. Cardiovascular Diagnosis and Therapy. 2020; 10(2): 350. DOI: 10.21037/cdt.2019.09.21 32420117 PMC7225444

[148] Agyepong IA, Sewankambo N, Binagwaho A, Coll-Seck AM, Corrah T, Ezeh A, et al. The path to longer and healthier lives for all Africans by 2030: The Lancet Commission on the future of health in sub-Saharan Africa. The Lancet. 2017; 390(10114): 2803–59. DOI: 10.1016/S0140-6736(17)31509-X 28917958

[149] The World Bank. Universal Health Coverage (UHC) in Africa: A Framework for Action: Main Report. The World Bank; 2016.

[150] Healey JS, Crystal E, Lamy A, Teoh K, Semelhago L, Hohnloser SH, et al. Left Atrial Appendage Occlusion Study (LAAOS): Results of a randomized controlled pilot study of left atrial appendage occlusion during coronary bypass surgery in patients at risk for stroke. Am Heart J. 2005; 150(2): 288–93. DOI: 10.1016/j.ahj.2004.09.054 16086933

[151] Whitlock RP, Vincent J, Blackall MH, Hirsh J, Fremes S, Novick R, et al. Left Atrial Appendage Occlusion Study II (LAAOS II). Can J Cardiol. 2013; 29(11): 1443–7. DOI: 10.1016/j.cjca.2013.06.015 24054920

[152] Tsai YC, Phan K, Munkholm-Larsen S, Tian DH, La Meir M, Yan TD. Surgical left atrial appendage occlusion during cardiac surgery for patients with atrial fibrillation: A meta-analysis. Eur J Cardiothorac Surg. 2015; 47(5): 847–54. DOI: 10.1093/ejcts/ezu291 25064051

[153] Holmes DR, Jr., Doshi SK, Kar S, Price MJ, Sanchez JM, Sievert H, et al. Left Atrial Appendage Closure as an Alternative to Warfarin for Stroke Prevention in Atrial Fibrillation: A Patient-Level Meta-Analysis. J Am Coll Cardiol. 2015; 65(24): 2614–23. DOI: 10.1016/j.jacc.2015.04.025 26088300

[154] Boersma LV, Ince H, Kische S, Pokushalov E, Schmitz T, Schmidt B, et al. Efficacy and safety of left atrial appendage closure with WATCHMAN in patients with or without contraindication to oral anticoagulation: 1-Year follow-up outcome data of the EWOLUTION trial. Heart Rhythm. 2017; 14(9): 1302–8. DOI: 10.1016/j.hrthm.2017.05.038 28577840

[155] Hicks T, Stewart F, Eisinga A. NOACs versus warfarin for stroke prevention in patients with AF: a systematic review and meta-analysis. Open heart. 2016; 3(1): e000279. DOI: 10.1136/openhrt-2015-000279 26848392 PMC4731839

[156] Laing R, Waning B, Gray A, Ford N, Hoen E. 25 years of the WHO essential medicines lists: progress and challenges. The Lancet. 2003; 361(9370): 1723–9. DOI: 10.1016/S0140-6736(03)13375-2 12767751

[157] Di Cesare M, Jarvis JD, Scarlatescu O, Leng X, Zaidel EJ, Burrone E, et al. NOACs Added to WHO’s Essential Medicines List: Recommendations for Future Policy Actions. Glob Heart. 2020; 15(1): 67. DOI: 10.5334/gh.774 33150132 PMC7546116

[158] Cowan JC, Wu J, Hall M, Orlowski A, West RM, Gale CP. A 10 year study of hospitalized atrial fibrillation-related stroke in England and its association with uptake of oral anticoagulation. Eur Heart J. 2018; 39(32): 2975–83. DOI: 10.1093/eurheartj/ehy411 29982405 PMC6110195

[159] Chao TF, Chiang CE, Lin YJ, Chang SL, Lo LW, Hu YF, et al. Evolving Changes of the Use of Oral Anticoagulants and Outcomes in Patients With Newly Diagnosed Atrial Fibrillation in Taiwan. Circulation. 2018; 138(14): 1485–7. DOI: 10.1161/CIRCULATIONAHA.118.036046 30354355

[160] Lee SR, Choi EK, Han KD, Cha MJ, Oh S, Lip GYH. Temporal trends of antithrombotic therapy for stroke prevention in Korean patients with non-valvular atrial fibrillation in the era of non-vitamin K antagonist oral anticoagulants: A nationwide population-based study. PLoS One. 2017; 12(12): e0189495. DOI: 10.1371/journal.pone.0189495 29261716 PMC5738023

[161] Wallentin L, Yusuf S, Ezekowitz MD, Alings M, Flather M, Franzosi MG, et al. Efficacy and safety of dabigatran compared with warfarin at different levels of international normalised ratio control for stroke prevention in atrial fibrillation: an analysis of the RE-LY trial. Lancet. 2010; 376(9745): 975–83. DOI: 10.1016/S0140-6736(10)61194-4 20801496

[162] Gamra H, Murin J, Chiang CE, Naditch-Brûlé L, Brette S, Steg PG. Use of antithrombotics in atrial fibrillation in Africa, Europe, Asia and South America: insights from the International RealiseAF Survey. Arch Cardiovasc Dis. 2014; 107(2): 77–87. DOI: 10.1016/j.acvd.2014.01.001 24556189

[163] Guo J, Guan T, Fan S, Chao B, Wang L, Liu Y. Underuse of Oral Anticoagulants in Patients With Ischemic Stroke and Atrial Fibrillation in China. Am J Cardiol. 2018; 122(12): 2055–61. DOI: 10.1016/j.amjcard.2018.08.057 30292336

[164] Chang SS, Dong JZ, Ma CS, Du X, Wu JH, Tang RB, et al. Current Status and Time Trends of Oral Anticoagulation Use Among Chinese Patients With Nonvalvular Atrial Fibrillation: The Chinese Atrial Fibrillation Registry Study. Stroke. 2016; 47(7): 1803–10. DOI: 10.1161/STROKEAHA.116.012988 27283198

[165] Karthikeyan G, Connolly SJ, Ntsekhe M, Benz A, Rangarajan S, Lewis G, et al. The INVICTUS rheumatic heart disease research program: Rationale, design and baseline characteristics of a randomized trial of rivaroxaban compared to vitamin K antagonists in rheumatic valvular disease and atrial fibrillation. Am Heart J. 2020; 225: 69–77. DOI: 10.1016/j.ahj.2020.03.018 32474206

[166] Guimarães HP, Lopes RD, de Barros ESPGM, Liporace IL, Sampaio RO, Tarasoutchi F, et al. Rivaroxaban in Patients with Atrial Fibrillation and a Bioprosthetic Mitral Valve. N Engl J Med. 2020; 383(22): 2117–26. DOI: 10.1056/NEJMoa2029603 33196155

[167] Meyer S, Frei CR, Daniels KR, Forcade NA, Bussey M, Bussey-Smith KL, et al. Impact of a new method of warfarin management on patient satisfaction, time, and cost. Pharmacotherapy. 2013; 33(11): 1147–55. DOI: 10.1002/phar.1344 24038425

[168] Barcellona D, Fenu L, Marongiu F. Point-of-care testing INR: An overview. Clin Chem Lab Med. 2017; 55(6): 800–5. DOI: 10.1515/cclm-2016-0381 27754958

[169] Singer DE, Hellkamp AS, Piccini JP, Mahaffey KW, Lokhnygina Y, Pan G, et al. Impact of global geographic region on time in therapeutic range on warfarin anticoagulant therapy: Data from the ROCKET AF clinical trial. J Am Heart Assoc. 2013; 2(1): e000067. DOI: 10.1161/JAHA.112.000067 23525418 PMC3603243

[170] Lowres N, Freedman B. Time to develop guidelines for screening and management of atrial fibrillation in Indigenous Australians. Med J Aust. 2020; 212(5): 212–3. DOI: 10.5694/mja2.50513 32083309

[171] Nedkoff L, Kelty EA, Hung J, Thompson SC, Katzenellenbogen JM. Differences in stroke risk and cardiovascular mortality for Aboriginal and other Australian patients with atrial fibrillation. Med J Aust. 2020; 212(5): 215–21. DOI: 10.5694/mja2.50496 32030754

[172] Gu Y, Doughty RN, Freedman B, Kennelly J, Warren J, Harwood M, et al. Burden of atrial fibrillation in Māori and Pacific people in New Zealand: A cohort study. Intern Med J. 2018; 48(3): 301–9. DOI: 10.1111/imj.13648 29034985

[173] Marcolino MS, Polanczyk CA, Bovendorp AC, Marques NS, Silva LA, Turquia CP, et al. Economic evaluation of the new oral anticoagulants for the prevention of thromboembolic events: A cost-minimization analysis. Sao Paulo Med J. 2016; 134(4): 322–9. DOI: 10.1590/1516-3180.2016.0019260216 27581333 PMC10876344

[174] Deshpande CG, Kogut S, Laforge R, Willey C. Impact of medication adherence on risk of ischemic stroke, major bleeding and deep vein thrombosis in atrial fibrillation patients using novel oral anticoagulants. Curr Med Res Opin. 2018; 34(7): 1285–92. DOI: 10.1080/03007995.2018.1428543 29334815

[175] Alberts MJ, Peacock WF, Fields LE, Bunz TJ, Nguyen E, Milentijevic D, et al. Association between once- and twice-daily direct oral anticoagulant adherence in nonvalvular atrial fibrillation patients and rates of ischemic stroke. Int J Cardiol. 2016; 215: 11–3. DOI: 10.1016/j.ijcard.2016.03.212 27104919

[176] Borne RT, O’Donnell C, Turakhia MP, Varosy PD, Jackevicius CA, Marzec LN, et al. Adherence and outcomes to direct oral anticoagulants among patients with atrial fibrillation: Findings from the veterans health administration. BMC Cardiovasc Disord. 2017; 17(1): 236. DOI: 10.1186/s12872-017-0671-6 28865440 PMC5581418

[177] Manzoor BS, Lee TA, Sharp LK, Walton SM, Galanter WL, Nutescu EA. Real-World Adherence and Persistence with Direct Oral Anticoagulants in Adults with Atrial Fibrillation. Pharmacotherapy. 2017; 37(10): 1221–30. DOI: 10.1002/phar.1989 28730619

[178] Maura G, Billionnet C, Alla F, Gagne JJ, Pariente A. Comparison of Treatment Persistence with Dabigatran or Rivaroxaban versus Vitamin K Antagonist Oral Anticoagulants in Atrial Fibrillation Patients: A Competing Risk Analysis in the French National Health Care Databases. Pharmacotherapy. 2018; 38(1): 6–18. DOI: 10.1002/phar.2046 29028119

[179] Mueller T, Alvarez-Madrazo S, Robertson C, Bennie M. Use of direct oral anticoagulants in patients with atrial fibrillation in Scotland: Applying a coherent framework to drug utilisation studies. Pharmacoepidemiol Drug Saf. 2017; 26(11): 1378–86. DOI: 10.1002/pds.4272 28752670 PMC5697642

[180] Rodriguez-Bernal CL, Peiro S, Hurtado I, Garcia-Sempere A, Sanfelix-Gimeno G. Primary Nonadherence to Oral Anticoagulants in Patients with Atrial Fibrillation: Real-World Data from a Population-Based Cohort. J Manag Care Spec Pharm. 2018; 24(5): 440–8. DOI: 10.18553/jmcp.2018.24.5.440 29694286 PMC10398152

[181] Emren SV, Senoz O, Bilgin M, Beton O, Aslan A, Taskin U, et al. Drug Adherence in Patients with Nonvalvular Atrial Fibrillation Taking Non-Vitamin K Antagonist Oral Anticoagulants in Turkey: NOAC-TR. Clin Appl Thromb Hemost. 2018; 24(3): 525–31. DOI: 10.1177/1076029617693940 28301907 PMC6714660

[182] Buchholz A, Ueberham L, Gorczynska K, Dinov B, Hilbert S, Dagres N, et al. Initial apixaban dosing in patients with atrial fibrillation. Clin Cardiol. 2018; 41(5): 671–6. DOI: 10.1002/clc.22949 29542830 PMC6489803

[183] Harper P, Pollock D, Stephens M. Dabigatran persistence and adherence in New Zealand: a nationwide retrospective observational study. BMJ Open. 2018; 8(4): e020212. DOI: 10.1136/bmjopen-2017-020212 PMC5892768 29626048

[184] Reading SR, Black MH, Singer DE, Go AS, Fang MC, Udaltsova N, et al. Risk factors for medication non-adherence among atrial fibrillation patients. BMC Cardiovasc Disord. 2019; 19(1): 38. DOI: 10.1186/s12872-019-1019-1 30744554 PMC6371431

[185] Gomez-Lumbreras A, Cortes J, Giner-Soriano M, Quijada-Manuitt MA, Morros R. Characteristics of Apixaban-Treated Patients, Evaluation of the Dose Prescribed, and the Persistence of Treatment: A Cohort Study in Catalonia. J Cardiovasc Pharmacol Ther. 2018; 23(6): 494–501. DOI: 10.1177/1074248418778544 29792125

[186] Nobili A, Marengoni A, Tettamanti M, Salerno F, Pasina L, Franchi C, et al. Association between clusters of diseases and polypharmacy in hospitalized elderly patients: Results from the REPOSI study. Eur J Intern Med. 2011; 22(6): 597–602. DOI: 10.1016/j.ejim.2011.08.029 22075287

[187] Noseworthy PA, Brito JP, Kunneman M, Hargraves IG, Zeballos-Palacios C, Montori VM, et al. Shared decision-making in atrial fibrillation: Navigating complex issues in partnership with the patient. J Interv Card Electrophysiol; 2018. DOI: 10.1007/s10840-018-0465-5 PMC7056296 30327992

[188] Pritchett RV, Bem D, Turner GM, Thomas GN, Clarke JL, Fellows R, et al. Improving the Prescription of Oral Anticoagulants in Atrial Fibrillation: A Systematic Review. Thromb Haemost. 2019; 119(2): 294–307. DOI: 10.1055/s-0038-1676835 30669165

[189] Martinez C, Wallenhorst C, Rietbrock S, Freedman B. Ischemic Stroke and Transient Ischemic Attack Risk Following Vitamin K Antagonist Cessation in Newly Diagnosed Atrial Fibrillation: A Cohort Study. J Am Heart Assoc. 2020; 9(2): e014376. DOI: 10.1161/JAHA.119.014376 31937194 PMC7033838

[190] Lowres N, Giskes K, Freedman B. Next frontier for stroke prevention in atrial fibrillation: Ensuring anticoagulant persistence. Heart; 2020. DOI: 10.1136/heartjnl-2020-318392 33361351

[191] Nutbeam D. The evolving concept of health literacy. Soc Sci Med. 2008; 67(12): 2072–8. DOI: 10.1016/j.socscimed.2008.09.050 18952344

[192] Lane DA, Meyerhoff J, Rohner U, Lip GYH. Atrial fibrillation patient preferences for oral anticoagulation and stroke knowledge: Results of a conjoint analysis. Clin Cardiol. 2018; 41(6): 855–61. DOI: 10.1002/clc.22971 29696664 PMC6489774

[193] Hoe R, Lin W, Bautista MAC, Vrijhoef HJM, Lim TW. Validation of a questionnaire measuring patient knowledge of atrial fibrillation in an Asian cohort. Heart Asia. 2019; 11(1): e011143. DOI: 10.1136/heartasia-2018-011143 31244915 PMC6560923

[194] Amara W, Larsen TB, Sciaraffia E, Hernández Madrid A, Chen J, Estner H, et al. Patients’ attitude and knowledge about oral anticoagulation therapy: results of a self-assessment survey in patients with atrial fibrillation conducted by the European Heart Rhythm Association. EP Europace. 2015; 18(1): 151–5. DOI: 10.1093/europace/euv317 26462697

[195] Seligman WH, Das-Gupta Z, Jobi-Odeneye AO, Arbelo E, Banerjee A, Bollmann A, et al. Development of an international standard set of outcome measures for patients with atrial fibrillation: a report of the International Consortium for Health Outcomes Measurement (ICHOM) atrial fibrillation working group. Eur Heart J. 2020; 41(10): 1132–40. DOI: 10.1093/eurheartj/ehz871 31995195 PMC7060456

[196] Arbelo E, Aktaa S, Bollmann A, D’Avila A, Drossart I, Dwight J, et al. Quality indicators for the care and outcomes of adults with atrial fibrillation. Europace; 2020. 10.1093/europace/euaa253 32860039

[197] Smet L, Heggermont WA, Goossens E, Eeckloo K, Vander Stichele R, De Potter T, et al. Adherence, knowledge, and perception about oral anticoagulants in patients with atrial fibrillation at high risk for thromboembolic events after radiofrequency ablation. J Adv Nurs. 2018; 74(11): 2577–87. DOI: 10.1111/jan.13780 29944735

[198] Maikranz V, Siebenhofer A, Ulrich L-R, Mergenthal K, Schulz-Rothe S, Kemperdick B, et al. Does a complex intervention increase patient knowledge about oral anticoagulation? A cluster-randomised controlled trial. BMC Fam Pract. 2017; 18(1): 15. DOI: 10.1186/s12875-017-0588-2 28166725 PMC5295216

[199] Desteghe L, Germeys J, Vijgen J, Koopman P, Dilling-Boer D, Schurmans J, et al. Effectiveness and usability of an online tailored education platform for atrial fibrillation patients undergoing a direct current cardioversion or pulmonary vein isolation. Int J Cardiol. 2018; 272: 123–9. DOI: 10.1016/j.ijcard.2018.07.065 30049498

[200] Marquez-Contreras E, Martell-Claros N, Marquez-Rivero S, Hermida-Campa E, Gracia-Diez C, Sanchez-Lopez E, et al. Strategies for improving dabigatran adherence for stroke prevention in patients with non-valvular atrial fibrillation: Education and drug intake reminders (FACILITA study). Curr Med Res Opin. 2018; 34(7): 1301–8. DOI: 10.1080/03007995.2018.1435519 29384410

[201] Vinereanu D, Lopes RD, Bahit MC, Xavier D, Jiang J, Al-Khalidi HR, et al. A multifaceted intervention to improve treatment with oral anticoagulants in atrial fibrillation (IMPACT-AF): An international, cluster-randomised trial. The Lancet. 2017; 390(10104): 1737–46. DOI: 10.1016/S0140-6736(17)32165-7 28859942

[202] Guo Y, Lane DA, Wang L, Zhang H, Wang H, Zhang W, et al. Mobile health technology to improve care for patients with atrial fibrillation. J Am Coll Cardiol. 2020; 75(13): 1523–34. DOI: 10.1016/j.jacc.2020.01.052 32241367

[203] Eckman MH, Costea A, Attari M, Munjal J, Wise RE, Knochelmann C, et al. Shared decision-making tool for thromboprophylaxis in atrial fibrillation – A feasibility study. Am Heart J. 2018; 199: 13–21. DOI: 10.1016/j.ahj.2018.01.003 29754650

[204] Stephan LS, Almeida ED, Guimaraes RB, Ley AG, Mathias RG, Assis MV, et al. Oral Anticoagulation in Atrial Fibrillation: Development and Evaluation of a Mobile Health Application to Support Shared Decision-Making. Arq Bras Cardiol. 2018; 110(1): 7–15. DOI: 10.5935/abc.20170181 29412241 PMC5831296

[205] Rolls CA, Obamiro KO, Chalmers L, Bereznicki LRE. The relationship between knowledge, health literacy, and adherence among patients taking oral anticoagulants for stroke thromboprophylaxis in atrial fibrillation. Cardiovasc Ther. 2017; 35(6). DOI: 10.1111/1755-5922.12304 28869793

[206] Heidbuchel H, Dagres N, Antz M, Kuck K-H, Lazure P, Murray S, et al. Major knowledge gaps and system barriers to guideline implementation among European physicians treating patients with atrial fibrillation: A European Society of Cardiology international educational needs assessment. Ep Europace. 2018; 20(12): 1919–28. DOI: 10.1093/europace/euy039 29538637

[207] Steffel J, Verhamme P, Potpara TS, Albaladejo P, Antz M, Desteghe L, et al. The 2018 European Heart Rhythm Association Practical Guide on the use of non-vitamin K antagonist oral anticoagulants in patients with atrial fibrillation: executive summary. EP Europace. 2018; 20(8): 1231–42. DOI: 10.1093/europace/euy054 29562331

[208] Orchard JJ, Neubeck L, Orchard JW, Puranik R, Raju H, Freedman B, et al. ECG-based cardiac screening programs: Legal, ethical, and logistical considerations. Heart Rhythm. 2019; 16(10): 1584–91. DOI: 10.1016/j.hrthm.2019.03.025 30930331

[209] Zhang L, Gallagher R, Neubeck L. Health-related quality of life in atrial fibrillation patients over 65 years: a review. Eur J Prev Cardiol. 2015; 22(8): 987–1002. DOI: 10.1177/2047487314538855 24924742

[210] Kirchhof P, Benussi S, Kotecha D, Ahlsson A, Atar D, Casadei B, et al. 2016 ESC Guidelines for the management of atrial fibrillation developed in collaboration with EACTS. Eur J Cardiothorac Surg. 2016; 50(5): e1–e88. 27663299 10.1093/ejcts/ezw313

[211] Kirchhof P. The future of atrial fibrillation management: Integrated care and stratified therapy. The Lancet. 2017; 390(10105): 1873–87. DOI: 10.1016/S0140-6736(17)31072-3 28460828

[212] Pastori D, Menichelli D, Violi F, Pignatelli P, G YHL. The Atrial fibrillation Better Care (ABC) pathway and cardiac complications in atrial fibrillation: A potential sex-based difference. The ATHERO-AF study. Eur J Intern Med; 2020. DOI: 10.1093/ehjci/ehaa946.0346 33358066

[213] Healey JS, Oldgren J, Ezekowitz M, Zhu J, Pais P, Wang J, et al. Occurrence of death and stroke in patients in 47 countries 1 year after presenting with atrial fibrillation: a cohort study. Lancet. 2016; 388(10050): 1161–9. DOI: 10.1016/S0140-6736(16)30968-0 27515684

[214] Schnabel RB, Yin X, Gona P, Larson MG, Beiser AS, McManus DD, et al. 50 year trends in atrial fibrillation prevalence, incidence, risk factors, and mortality in the Framingham Heart Study: A cohort study. The Lancet. 2015; 386(9989): 154–62. DOI: 10.1016/S0140-6736(14)61774-8 PMC4553037 25960110

[215] Rahman F, Wang N, Yin X, Ellinor PT, Lubitz SA, LeLorier PA, et al. Atrial flutter: Clinical risk factors and adverse outcomes in the Framingham Heart Study. Heart Rhythm. 2016; 13(1): 233–40. DOI: 10.1016/j.hrthm.2015.07.031 26226213 PMC4698205

[216] Chung MK, Eckhardt LL, Chen LY, Ahmed HM, Gopinathannair R, Joglar JA, et al. Lifestyle and Risk Factor Modification for Reduction of Atrial Fibrillation: A Scientific Statement From the American Heart Association. Circulation. 2020; 141(16): e750–e72. DOI: 10.1161/CIR.0000000000000748 32148086

[217] Wong JA, Conen D, Van Gelder IC, McIntyre WF, Crijns HJ, Wang J, et al. Progression of Device-Detected Subclinical Atrial Fibrillation and the Risk of Heart Failure. J Am Coll Cardiol. 2018; 71(23): 2603–11. DOI: 10.1016/j.jacc.2018.03.519 29880119

[218] Roy D, Talajic M, Nattel S, Wyse DG, Dorian P, Lee KL, et al. Rhythm control versus rate control for atrial fibrillation and heart failure. N Engl J Med. 2008; 358(25): 2667–77. DOI: 10.1056/NEJMoa0708789 18565859

[219] Marrouche NF, Brachmann J, Andresen D, Siebels J, Boersma L, Jordaens L, et al. Catheter Ablation for Atrial Fibrillation with Heart Failure. N Engl J Med. 2018; 378(5): 417–27. DOI: 10.1056/NEJMoa1707855 29385358

[220] Gaita F, Leclercq JF, Schumacher B, Scaglione M, Toso E, Halimi F, et al. Incidence of silent cerebral thromboembolic lesions after atrial fibrillation ablation may change according to technology used: comparison of irrigated radiofrequency, multipolar nonirrigated catheter and cryoballoon. J Cardiovasc Electrophysiol. 2011; 22(9): 961–8. DOI: 10.1111/j.1540-8167.2011.02050.x 21453372

[221] Conen D, Rodondi N, Müller A, Beer JH, Ammann P, Moschovitis G, et al. Relationships of Overt and Silent Brain Lesions with Cognitive Function in Patients with Atrial Fibrillation. J Am Coll Cardiol. 2019; 73(9): 989–99. DOI: 10.1016/j.jacc.2018.12.039 30846109

[222] Thacker EL, McKnight B, Psaty BM, Longstreth WT, Jr., Sitlani CM, Dublin S, et al. Atrial fibrillation and cognitive decline: A longitudinal cohort study. Neurology. 2013; 81(2): 119–25. DOI: 10.1212/WNL.0b013e31829a33d1 23739229 PMC3770176

[223] Lopes RD, Alings M, Connolly SJ, Beresh H, Granger CB, Mazuecos JB, et al. Rationale and design of the Apixaban for the Reduction of Thrombo-Embolism in Patients with Device-Detected Sub-Clinical Atrial Fibrillation (ARTESiA) trial. Am Heart J. 2017; 189: 137–45. DOI: 10.1016/j.ahj.2017.04.008 28625370

[224] Rivard L, Khairy P, Talajic M, Tardif JC, Nattel S, Bherer L, et al. Blinded Randomized Trial of Anticoagulation to Prevent Ischemic Stroke and Neurocognitive Impairment in Atrial Fibrillation (BRAIN-AF): Methods and Design. Can J Cardiol. 2019; 35(8): 1069–77. DOI: 10.1016/j.cjca.2019.04.022 31376908

[225] Claes N, Van Laethem C, Goethals M, Goethals P, Mairesse G, Schwagten B, et al. Prevalence of atrial fibrillation in adults participating in a large-scale voluntary screening programme in Belgium. Acta Cardiol. 2012; 67(3): 273–8. DOI: 10.1080/AC.67.3.2160714 22870733

[226] Zhou Z, Hu D. An Epidemiological Study on the Prevalence of Atrial Fibrillation in the Chinese Population of Mainland China. J Epidemiol. 2008; 18(5): 209–16. DOI: 10.2188/jea.JE2008021 18776706 PMC4771592

[227] Wang Z, Chen Z, Wang X, Zhang L, Li S, Tian Y, et al. The Disease Burden of Atrial Fibrillation in China from a National Cross-sectional Survey. Am J Cardiol. 2018; 122(5): 793–8. DOI: 10.1016/j.amjcard.2018.05.015 30049467

[228] Fitzmaurice DA, Hobbs FDR, Jowett S, Mant J, Murray ET, Holder R, et al. Screening versus routine practice in detection of atrial fibrillation in patients aged 65 or over: Cluster randomised controlled trial. BMJ (Clinical research ed). 2007; 335(7616): 383. DOI: 10.1136/bmj.39280.660567.55 PMC1952508 17673732

[229] Davis RC, Hobbs FDR, Kenkre JE, Roalfe AK, Iles R, Lip GYH, et al. Prevalence of atrial fibrillation in the general population and in high-risk groups: the ECHOES study. EP Europace. 2012; 14(11): 1553–9. DOI: 10.1093/europace/eus087 22490371

[230] Schnabel RB, Wilde S, Wild PS, Munzel T, Blankenberg S. Atrial fibrillation: Its prevalence and risk factor profile in the German general population. Deutsches Ärzteblatt International. 2012; 109(16): 293. DOI: 10.3238/arztebl.2012.0293 22577476 PMC3349225

[231] Bilato C, Corti M-C, Baggio G, Rampazzo D, Cutolo A, Iliceto S, et al. Prevalence, Functional Impact, and Mortality of Atrial Fibrillation in an Older Italian Population (from the Pro.V.A. Study). Am J Cardiol. 2009; 104(8): 1092–7. DOI: 10.1016/j.amjcard.2009.05.058 19801031

[232] Di Carlo A, Bellino L, Consoli D, Mori F, Zaninelli A, Baldereschi M, et al. Prevalence of atrial fibrillation in the Italian elderly population and projections from 2020 to 2060 for Italy and the European Union: The FAI Project. Europace. 2019; 21(10): 1468–75. DOI: 10.1093/europace/euz141 31131389

[233] Bonhorst D, Mendes M, Adragão P, De Sousa J, Primo J, Leiria E, et al. Prevalence of atrial fibrillation in the Portuguese population aged 40 and over: The FAMA study. Rev Port Cardiol. 2010; 29(3): 331–50. 20635561

[234] Monteiro P. The SAFIRA study: A reflection on the prevalence and treatment patterns of atrial fibrillation and cardiovascular risk factors in 7500 elderly subjects. Revista Portuguesa de Cardiologia (English edition). 2018; 37(4): 307–13. DOI: 10.1016/j.repce.2017.08.006 29703518

[235] Gómez-Doblas JJ, Muñiz J, Martin JJA, Rodríguez-Roca G, Lobos JM, Awamleh P, et al. Prevalence of Atrial Fibrillation in Spain. OFRECE Study Results. Revista Española de Cardiología (English Edition). 2014; 67(4): 259–69. DOI: 10.1016/j.rec.2013.07.014 24774588

[236] Joan Sala RM, Marrugat J, Pena A. Prevalencia de fibrilación auricular en la provincia de Girona: el Estudio REGICOR. Rev Esp Cardiol. 2001; 54(10): 1240. DOI: 10.1016/S0300-8932(01)76486-X 11591309

[237] Engdahl J, Andersson L, Mirskaya M, Rosenqvist M. Stepwise Screening of Atrial Fibrillation in a 75-Year-Old Population. Circulation. 2013; 127(8): 930–7. DOI: 10.1161/CIRCULATIONAHA.112.126656 23343564

[238] Andersson P, Löndahl M, Abdon N-J, Terent A. The prevalence of atrial fibrillation in a geographically well-defined population in Northern Sweden: implications for anticoagulation prophylaxis. J Intern Med. 2012; 272(2): 170–6. DOI: 10.1111/j.1365-2796.2012.02519.x 22250988

[239] Koopman JJE, van Bodegom D, Westendorp RGJ, Jukema JW. Scarcity of atrial fibrillation in a traditional African population: A community-based study. BMC Cardiovasc Disord. 2014; 14(1): 87. DOI: 10.1186/1471-2261-14-87 25037974 PMC4107622

[240] Dewhurst MJ, Adams PC, Gray WK, Dewhurst F, Orega GP, Chaote P, et al. Strikingly low prevalence of atrial fibrillation in elderly Tanzanians. J Am Geriatr Soc. 2012; 60(6): 1135–40. DOI: 10.1111/j.1532-5415.2012.03963.x 22646732

[241] Tegene E, Tadesse I, Markos Y, Gobena T. Prevalence and risk factors for atrial fibrillation and its anticoagulant requirement in adults aged ≥40 in Jimma Town, Southwest Ethiopia: A community based cross-sectional study. Int J Cardiol Heart Vasc. 2019; 22: 199–204. DOI: 10.1016/j.ijcha.2019.02.003 30963095 PMC6437289

[242] Allan V, Honarbakhsh S, Casas JP, Wallace J, Hunter R, Schilling R, et al. Are cardiovascular risk factors also associated with the incidence of atrial fibrillation? A systematic review and field synopsis of 23 factors in 32 population-based cohorts of 20 million participants. Thromb Haemost. 2017; 117(5): 837–50. DOI: 10.1160/TH16-11-0825 28229164 PMC5442605

[243] Benjamin EJ, Muntner P, Alonso A, Bittencourt MS, Callaway CW, Carson AP, et al. Heart Disease and Stroke Statistics-2019 Update: A Report From the American Heart Association. Circulation. 2019; 139(10): e56–e528. 30700139 10.1161/CIR.0000000000000659

[244] Kirchhof P, Lip GY, Van Gelder IC, Bax J, Hylek E, Kaab S, et al. Comprehensive risk reduction in patients with atrial fibrillation: Emerging diagnostic and therapeutic options—A report from the 3rd Atrial Fibrillation Competence NETwork/European Heart Rhythm Association consensus conference. Europace. 2012; 14(1): 8–27. DOI: 10.1093/europace/eur241 21791573 PMC3236658

[245] Chang SH, Kuo CF, Chou IJ, See LC, Yu KH, Luo SF, et al. Association of a Family History of Atrial Fibrillation with Incidence and Outcomes of Atrial Fibrillation: A Population-Based Family Cohort Study. JAMA Cardiol. 2017; 2(8): 863–70. DOI: 10.1001/jamacardio.2017.1855 28678986 PMC5710587

[246] Fox CS, Parise H, D’Agostino RB, Sr., Lloyd-Jones DM, Vasan RS, Wang TJ, et al. Parental atrial fibrillation as a risk factor for atrial fibrillation in offspring. JAMA. 2004; 291(23): 2851–5. DOI: 10.1001/jama.291.23.2851 15199036

[247] Lubitz SA, Yin X, Fontes JD, Magnani JW, Rienstra M, Pai M, et al. Association between familial atrial fibrillation and risk of new-onset atrial fibrillation. JAMA. 2010; 304(20): 2263–9. DOI: 10.1001/jama.2010.1690 21076174 PMC3073054

[248] Zoller B, Ohlsson H, Sundquist J, Sundquist K. High familial risk of atrial fibrillation/atrial flutter in multiplex families: a nationwide family study in Sweden. J Am Heart Assoc. 2012; 2(1): e003384. DOI: 10.1161/JAHA.112.003384 23525409 PMC3603261

[249] Giacomantonio NB, Bredin SS, Foulds HJ, Warburton DE. A systematic review of the health benefits of exercise rehabilitation in persons living with atrial fibrillation. Can J Cardiol. 2013; 29(4): 483–91. DOI: 10.1016/j.cjca.2012.07.003 23200094

[250] Andersen K, Farahmand B, Ahlbom A, Held C, Ljunghall S, Michaelsson K, et al. Risk of arrhythmias in 52 755 long-distance cross-country skiers: A cohort study. Eur Heart J. 2013; 34(47): 3624–31. DOI: 10.1093/eurheartj/eht188 23756332

[251] Shamloo AS, Dagres N, Arya A, Hindricks G. Atrial fibrillation: A review of modifiable risk factors and preventive strategies. Rom J Intern Med. 2019; 57(2): 99–109. DOI: 10.2478/rjim-2018-0045 30648669

[252] Furberg CD, Psaty BM, Manolio TA, Gardin JM, Smith VE, Rautaharju PM. Prevalence of atrial fibrillation in elderly subjects (the Cardiovascular Health Study). Am J Cardiol. 1994; 74(3): 236–41. DOI: 10.1016/0002-9149(94)90363-8 8037127

[253] Santhanakrishnan R, Wang N, Larson MG, Magnani JW, McManus DD, Lubitz SA, et al. Atrial Fibrillation Begets Heart Failure and Vice Versa: Temporal Associations and Differences in Preserved Versus Reduced Ejection Fraction. Circulation. 2016; 133(5): 484–92. DOI: 10.1161/CIRCULATIONAHA.115.018614 26746177 PMC4738087

[254] Lip GYH, Collet JP, de Caterina R, Fauchier L, Lane DA, Larsen TB, et al. Antithrombotic Therapy in Atrial Fibrillation Associated with Valvular Heart Disease: Executive Summary of a Joint Consensus Document from the European Heart Rhythm Association (EHRA) and European Society of Cardiology Working Group on Thrombosis, Endorsed by the ESC Working Group on Valvular Heart Disease, Cardiac Arrhythmia Society of Southern Africa (CASSA), Heart Rhythm Society (HRS), Asia Pacific Heart Rhythm Society (APHRS), South African Heart (SA Heart) Association and Sociedad Latinoamericana de Estimulacion Cardiaca y Electrofisiologia (SOLEACE). Thromb Haemost. 2017; 117(12): 2215–36. DOI: 10.1160/TH-17-10-0709 29212110

[255] Benjamin EJ, Levy D, Vaziri SM, D’Agostino RB, Belanger AJ, Wolf PA. Independent risk factors for atrial fibrillation in a population-based cohort. The Framingham Heart Study. JAMA. 1994; 271(11): 840–4. DOI: 10.1001/jama.1994.03510350050036 8114238

[256] Loomba RS, Buelow MW, Aggarwal S, Arora RR, Kovach J, Ginde S. Arrhythmias in Adults with Congenital Heart Disease: What Are Risk Factors for Specific Arrhythmias? Pacing Clin Electrophysiol. 2017; 40(4): 353–61. DOI: 10.1111/pace.12983 27987225

[257] Alonso A, Jensen PN, Lopez FL, Chen LY, Psaty BM, Folsom AR, et al. Association of sick sinus syndrome with incident cardiovascular disease and mortality: the Atherosclerosis Risk in Communities study and Cardiovascular Health Study. PLoS One. 2014; 9(10): e109662. DOI: 10.1371/journal.pone.0109662 25285853 PMC4186847

[258] Aune D, Feng T, Schlesinger S, Janszky I, Norat T, Riboli E. Diabetes mellitus, blood glucose and the risk of atrial fibrillation: A systematic review and meta-analysis of cohort studies. J Diabetes Complications. 2018; 32(5): 501–11. DOI: 10.1016/j.jdiacomp.2018.02.004 29653902

[259] Alonso A, Lopez FL, Matsushita K, Loehr LR, Agarwal SK, Chen LY, et al. Chronic kidney disease is associated with the incidence of atrial fibrillation: the Atherosclerosis Risk in Communities (ARIC) study. Circulation. 2011; 123(25): 2946–53. DOI: 10.1161/CIRCULATIONAHA.111.020982 21646496 PMC3139978

[260] Bansal N, Zelnick LR, Alonso A, Benjamin EJ, de Boer IH, Deo R, et al. eGFR and Albuminuria in Relation to Risk of Incident Atrial Fibrillation: A Meta-Analysis of the Jackson Heart Study, the Multi-Ethnic Study of Atherosclerosis, and the Cardiovascular Health Study. Clin J Am Soc Nephrol. 2017; 12(9): 1386–98. DOI: 10.2215/CJN.01860217 28798221 PMC5586568

[261] Asad Z, Abbas M, Javed I, Korantzopoulos P, Stavrakis S. Obesity is associated with incident atrial fibrillation independent of gender: A meta-analysis. J Cardiovasc Electrophysiol. 2018; 29(5): 725–32. DOI: 10.1111/jce.13458 29443438

[262] Aune D, Sen A, Schlesinger S, Norat T, Janszky I, Romundstad P, et al. Body mass index, abdominal fatness, fat mass and the risk of atrial fibrillation: A systematic review and dose-response meta-analysis of prospective studies. Eur J Epidemiol. 2017; 32(3): 181–92. DOI: 10.1007/s10654-017-0232-4 28194602 PMC5380695

[263] Desai R, Patel U, Singh S, Bhuva R, Fong HK, Nunna P, et al. The burden and impact of arrhythmia in chronic obstructive pulmonary disease: Insights from the National Inpatient Sample. Int J Cardiol. 2019; 281: 49–55. DOI: 10.1016/j.ijcard.2019.01.074 30711267

[264] Steffel J, Verhamme P, Potpara TS, Albaladejo P, Antz M, Desteghe L, et al. The 2018 European Heart Rhythm Association Practical Guide on the use of non-vitamin K antagonist oral anticoagulants in patients with atrial fibrillation. Eur Heart J. 2018; 39(16): 1330–93. DOI: 10.1093/eurheartj/ehy136 29562325

